# Through the fog: Systematic review and meta-analysis of the prevalence and associated factors of poor post-operative visual outcome of cataract surgery in Sub-Saharan Africa

**DOI:** 10.1371/journal.pone.0315263

**Published:** 2024-12-09

**Authors:** Zufan Alamrie Asmare, Beminate Lemma Seifu, Bezawit Melak Fente, Yohannes Mekuria Negussie, Hiwot Altaye Asebe, Meklit Melaku Bezie, Mamaru Melkam, Angwach Abrham Asnake

**Affiliations:** 1 Department of Ophthalmology, School of Medicine and Health Science, Debre Tabor University, Debre Tabor, Ethiopia; 2 Department of Public Health, College of Medicine and Health Science, Samara University, Afar, Ethiopia; 3 Department of General Midwifery, School of Midwifery, College of Medicine & Health Sciences, University of Gondar, Gondar, Ethiopia; 4 Department of Medicine, Adama General Hospital and Medical College, Adama, Ethiopia; 5 Department of Public Health Officer, Institute of Public Health, College of Medicine and Health Sciences, University of Gondar, Gondar, Ethiopia; 6 Department of Psychiatry, College of Medicine & Health Sciences, University of Gondar, Gondar, Ethiopia; 7 Department of Epidemiology and Biostatistics, School of Public Health, College of Medicine and Health Sciences, Wolayita Sodo University, Soddom, Ethiopia; Eye Foundation Hospital / Eye Foundation Retina Institute, NIGERIA

## Abstract

**Background:**

Cataract, despite being treatable, persists to have a devastating impact on people’s health and livelihoods all over the world. In Sub-Saharan Africa (SSA), 1.7 million people are blind and 6.94 million are visually impaired due to cataract. Also, Cataract surgery outcomes remain below the World Health Organization (WHO) recommendations in SSA. Hence, this review aimed to estimate the pooled prevalence and associated factors of poor post-operative visual outcome in SSA.

**Method:**

An intensive literature search was performed from PubMed, Google Scholar, EMBASE, HINARI, Scopus, and Web of Sciences. Data were extracted by using a pre-tested and standardized data extraction format and analyzed by using STATA 17 statistical software. I^2^ tests to appraise the heterogeneity across the included studies, a random-effect model to estimate the pooled prevalence, and a sub-group analysis to discern the viable source of heterogeneity were executed. Potential publication bias was also assessed by funnel plot, Egger’s weighted correlation, and Begg’s regression. The odds ratio with its 95% confidence was used to reckon the association between the prevalence and factors.

**Result:**

From 201 identified studies, 25 articles were included. The pooled prevalence of poor post-operative visual outcome of cataract surgery in SSA was 14.56% (95% CI 11.31, 17.81). The presence of intra-operative complications (AOR = 2.99, 95% CI: 1.79, 4.98) and the presence of post-operative complications (AOR = 3.56, 95% CI: 2.86, 4.43) were statistically significant with the pooled poor post-operative visual outcome. According to the subgroup analysis, the pooled prevalence of poor post-operative visual outcome was found lower in phacoemulsification, with a sub-pooled prevalence of 12.32% (95% CI 7.89, 16.74) compared to incisional with a sub-pooled prevalence of 16.28% (95% CI 10.98, 21.59).

**Conclusion:**

This meta-analysis revealed that a substantial proportion of cataract-operated patients had poor post-operative visual outcome. The presence of intra-operative complications and post-operative complications were independent predictors of poor post-operative visual outcome. Therefore, improvement of post-operative visual outcome through decreasing intra-operative complications, managing post-operative complications, and investing in specialized training and equipment for ophthalmic surgeons are pivotal and need significant emphasis.

## Introduction

Cataract, despite being treatable, persists to have a devastating impact on people’s health and livelihoods all over the world [1]. Globally, It is the leading cause of blindness and the second leading cause of visual impairment (VI) [2]. Around 43.3 million people in the world are blind and 295 million people are visually impaired [3]. Of these 17 million (39.26%) people were blind and 83.5 million (28.3%) were visually impaired due to cataract [3–5]. It is particularly alarming to note that 89% of those affected live in low- and middle-income countries [5]. In addition, this problem is especially prevalent among older adults who may already require assistance due to age-related factors, making it more challenging to maintain a good quality of life and autonomy [6,7].

Individuals suffering from cataracts face a multitude of challenges, affecting their daily activities, independence, and quality of life [8]. The difficulty of simple tasks leads to decreased autonomy and increased reliance on others [8,9]. Globally, 338.3 million people live with blindness or visual impairment, nearly half of which could have been avoided through timely intervention [2,3]. Cataract is responsible for a combined total of 105 million cases of visual impairment and blindness [3]. In Sub-Saharan Africa (SSA), approximately 4.28 million individuals are blind, and 17.36 million are visually impaired. Among these, cataract is responsible for 1.7 million cases of blindness, accounting for about 39.71% of the regional blindness, and 6.94 million cases of visual impairment, representing approximately 39.97% of the regional visual impairment [10].

Visual rehabilitation involves mainly surgical interventions aimed at restoring sight. It is necessary to have proficient skills in cataract surgery and surgical initiatives to mitigate cataract-related blindness [11]. According to the World Health Organization (WHO), ≥80% of the operated eyes should have a presenting visual acuity of 6/6 to 6/18 following surgery, whereas less than 5% should have less than 6/60 [12]. After best correction, WHO recommends that >90% of cataract-operated eyes should have a visual acuity (VA) of ≥6/18, <5% with VA of <6/18-6/60 and <5% with VA <6/60 [13]. Yet, cataract surgery outcomes in sub-Saharan countries remain below WHO recommendations [12].

Post-operative visual outcome of cataract surgery is a key measure of the procedure’s effectiveness, yet these outcomes can differ widely across regions [14]. The prevalence of poor visual outcomes after cataract surgery is approximately 4% in the United States [15], 5.7% in Europe [16], and 16.6% in India [17]. While in SSA, this variation is more pronounced, ranging from as high as 59.8% in Jimma, Ethiopia [18], to as low as 1.8% in the eastern region of Ghana [19]. Evidence suggests that intra-operative and post-operative complications, ocular comorbidities, and the presence of systemic diseases increase the magnitude of poor post-operative visual outcomes [20].

Although independent studies were conducted on the poor post-operative visual outcome of cataract surgery and associated factors in SSA, there was a tremendous deviation and discordancy of the findings among the studies. As a result of this variability across studies, a pooled prevalence of poor visual outcome is needed. Besides, the inclusive estimate of the prevalence is essential in tackling the region’s penury for budding policies and bolstering programs that address the unique challenges posed by post-surgical visual impairment. Moreover, there is no systematically conducted representative study tackling this issue. Hence, we conducted this systematic review and meta-analysis with the aim of estimating the pooled prevalence of poor post-operative visual outcomes and associated factors in SSA.

## Materials and methods

### Design and searching strategies

To identify potentially relevant articles, we searched Google Scholar, Web of Science, PubMed/MEDLINE, Science Direct, and EMBASE exhaustively. All studies related to the poor visual outcome of cataract surgery and/or its associated factors in SSA were retrieved. Using references from retrieved articles and related systematic reviews, we conducted a manual search for additional relevant studies. The thorough examination of articles took place from February 1 to 29, 2024, using specific search combinations. The search terms were “magnitude”; “prevalence”; “proportion”; “visual outcome of cataract surgery”; “post-operative visual outcome”; “Adult”; “associated factor”; “determinants” and Sub-Saharan Africa. Separate searches were conducted for Boolean operator terms and together accordingly as “OR” or “AND” or AND NOT or AND, NOT. For reporting, we followed the Preferred Reporting Items for Systematic Reviews and Meta-Analyses (PRISMA) 2020 guidelines [21] (S1 File). The protocol was registered in the International Prospective Register of Systematic Reviews (PROSPERO) with registration number CRD42024590934.

### Eligible criteria

#### Inclusion criteria

Study area: Only those studies that were carried out in SSA were included.

Study design: All observational studies reporting the prevalence and associated factors of poor post-operative visual outcome of cataract surgery among older adults (>40 years) in SSA were eligible.

Language: Articles written only in the English language were considered.

Population: Studies done among older adults (>40 years) were considered.

Publication year: All research reports published from January 1, 2011 to February 29, 2024, were included.

#### Exclusion criteria

Studies whose 6–8 weeks post-operative visual acuities were not recorded, cataract with pre-existing ocular comorbidities such as glaucoma, age-related macular degeneration, corneal opacity, and systemic health problems such as diabetes mellitus and hypertension. Also, traumatic cataract, combined surgery cases, and those with difficult access to abstract and/or full text were excluded.

### Outcome measures

This systematic review and meta-analysis had two main objectives. The first objective was to determine the pooled prevalence of poor post-operative visual outcome of cataract surgery in sub-Saharan Africa. The surgical techniques used in the studies reviewed were either incisional or phacoemulsification. In the incisional category, the techniques included were Extracapsular Cataract Extraction (ECCE), Intracapsular Cataract Extraction (ICCE), and Small Incision Cataract Surgery (SICS). During the 6–8 weeks postoperative period, all participants underwent refraction to determine their final postoperative best-corrected visual acuity (BCVA). The visual outcomes were categorized based on the WHO classification of postoperative visual acuity outcomes [22]. The study applied the WHO criteria for cataract surgery outcomes as follows:

- Good Outcome: BCVA of 6/18 or better (≥ 6/18).
- Borderline Outcome: BCVA between <6/18 and 6/60.
- Poor Outcome: BCVA worse than 6/60 (<6/60).

Following the generation of prevalence and standard error of poor post-operative visual outcome, the pooled prevalence of poor post-operative visual outcome was calculated using the meta-prevalence standard error command.

The second objective was to identify factors associated with poor post-operative visual outcomes of cataract surgery in SSA.

Intraoperative complications are adverse events that occur during the surgical process, potentially altering the desired outcome and requiring corrective measures. Common examples include posterior capsule rupture, intra-operative hemorrhage, vitreous loss, zonular dehiscence, and retrobulbar hemorrhage among others [23].

Postoperative complications of cataract surgery refer to adverse conditions arising after the surgical procedure that may affect recovery or vision. These complications can include infections, inflammation (like uveitis, vitritis, endophthalmitis, and inflammatory cystoid macular edema), posterior capsule opacification, retinal detachment, and increased intraocular pressure among others [24].

### Quality assessment and data extraction

Two independent authors evaluated the quality of the included studies using the Joanna Briggs Institute (JBI) quality appraisal checklist. Studies that received a final quality rating checklist score of 50% or higher were then included. The retrieved studies from the databases were imported into Endnote citation manager version X9 for Windows, and duplicate articles were removed using reference management software (Endnote version X7.2) and manually. All papers were reviewed by three independent reviewers (AYG, GAA, and KEH): first, the abstracts and titles, then the full texts. Using a standardized Excel data extraction format, three independent investigators extracted data (ZAA, EYE, and YSA). In the spreadsheet; the first author’s name, publication year, Age (minimum age of study participants), study design, country, economic level of countries, sample size, pre-operative astigmatism, type of cataract surgery, intraoperative complications, postoperative complications (including post-operative inflammations such as uveitis, vitritis, endophthalmitis, and inflammatory cystoid macular edema, as well as other complications like posterior capsular opacifications among others), and postoperative BCVA were included (S2 File). Discussions among the reviewers were used to resolve disagreements.

### Data analysis

For analysis, the elicited data were exported from a Microsoft Excel spreadsheet to STATA version 17. Heterogeneity among the rooted studies was anthropometrically measured by an index of heterogeneity (I^2^ statistics), in which 25%, 50%, and 75% typify low, moderate, and high heterogeneity, respectively. Since the test statistics exhibited a considerable heterogeneity between studies (I^2^ = 98.51%, p<0.01), a random effect model was used to estimate the pooled prevalence. Subgroup analysis was performed based on the type of cataract surgery and the economic level of countries using the World Bank’s list of economies. However, statistically significant heterogeneity was only observed by the type of cataract surgery. Potential publication bias was also assessed subjectively by funnel plot and objectively using Begg’s and Egger’s tests at a 5% significance level. The odds ratio with its 95% confidence was used to assess the association between poor post-operative visual outcomes and factors. The results were offered as text and Forest plots.

## Result

### Searching results

**Fig 1:**
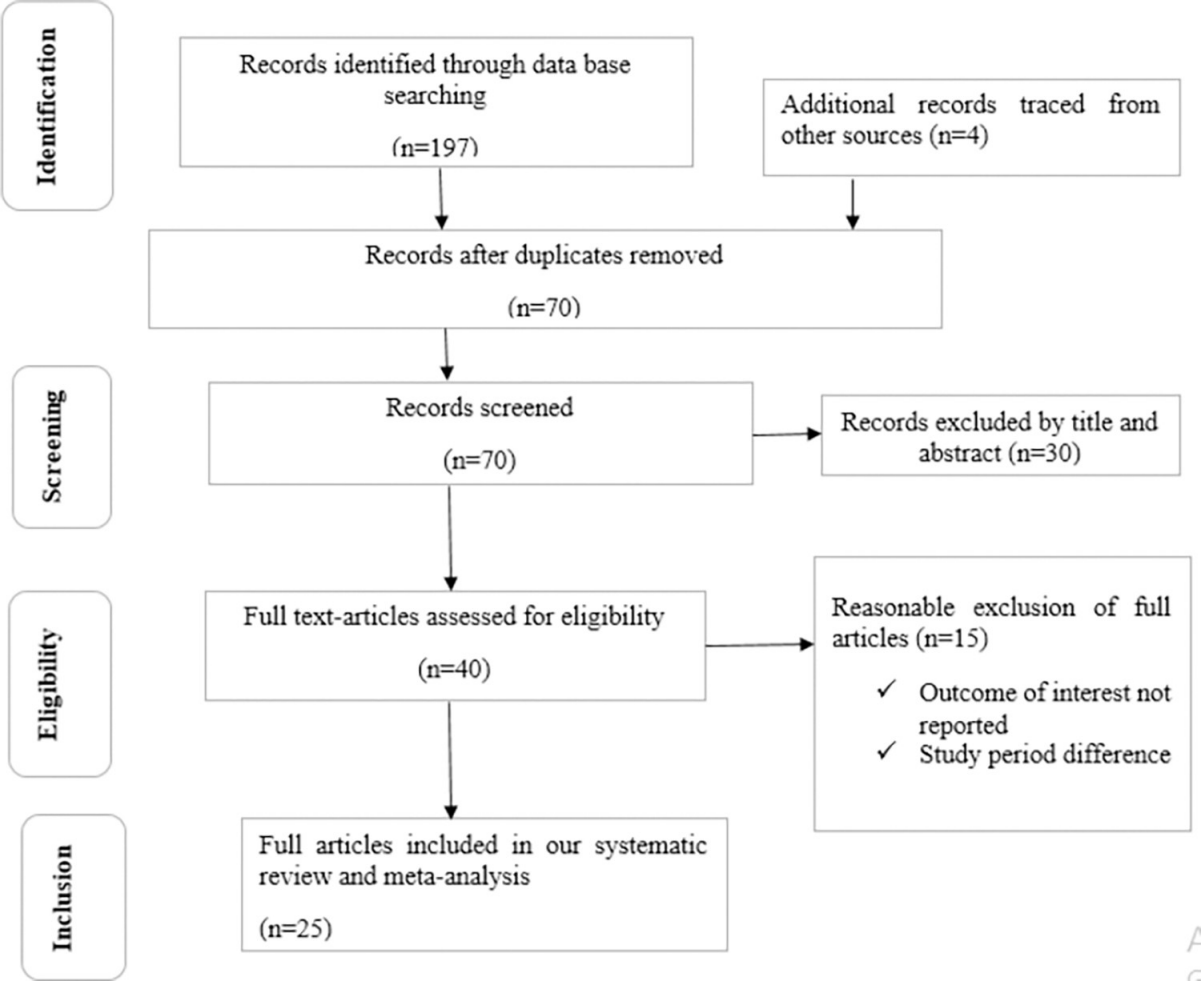
Flow chart diagram describing the selection of studies for the systematic review and meta-analysis of the poor post-operative visual outcome of cataract surgery and associated factors in Sub-Saharan Africa.

In total, 201 studies about the visual outcome of cataract surgery in SSA were identified through electronic searches, 197 through database searches, and 4 through other sources (academic library repositories) (S1 Table). Due to duplication, 131 studies were removed from this pool. Based on reviewing the titles and abstracts of the remaining 70 studies, 30 were excluded as they were not relevant to this systematic review and meta-analysis. The remaining 40 full-text articles were assessed for eligibility based on the eligibility criteria. Of these, 15 articles were further excluded for the following reasons (outcome of interest not reported, published before 2011, and different study populations). Finally, 25 studies achieved the eligibility criteria and were included in this systematic review and meta-analysis (Fig 1).

### Description of the included studies

A total of twenty-five studies were included in this systematic review and meta-analysis. A total of 11,556 adults aged 40 and older who had undergone cataract surgery were included in the study to estimate the pooled prevalence of poor post-operative visual outcomes. The publication period spanned from 2011 to 2024. The minimum and maximum sample sizes were 116 and 1540 in Nigeria and Ghana, respectively. Three of the included studies were from Ethiopia; 3 were from Ghana; 2 from Kenya; 4 from Nigeria; 2 from Cameroon; 2 from Liberia; 2 from Rwanda and 7 were from Mozambique, Togo, Tanzania, South Africa, Sudan, Zambia and Gabon. All the included studies had a quality score of 50% or higher (Table 1).

**Table 1:** Description of studies included in this systematic review and meta-analysis.

Author	Publication year	Country	Study Settings	Sample size	Prevalence of PPVO (%)	Quality Score (%)
Markos et al[25]	2020	Ethiopia	Facility based	322	11.50	75.00
Hussen et al[26]	2017	Ethiopia	Facility based	323	44.50	75.00
Mohammed et al[18]	2023	Ethiopia	Facility based	386	59.80	87.50
Danso et al.[19]	2022	Eastern Ghana	Facility based	447	1.80	87.50
Llechie et al.[27]	2012	Southern Ghana	Facility based	1288	9.50	75.00
Mangi et al[28]	2022	Karachi, Ghana	Facility based	1540	3.00	87.50
Rathi et al.[29]	2021	Liberia	Facility based	570	5.22	87.50
Javaloy et al.[30]	2021	Kenya	Facility based	849	6.30	75.00
Hashim et al.[31]	2013	Kenya	Facility based	495	2.20	75.00
Khanna et al.[32]	2020	Liberia	Facility based	575	5.22	75.00
Beyiah et al.[33]	2016	Cameroon	Facility based	230	33.3	75.00
Sengo et al.[34]	2023	Mozambique	Facility based	447	2.20	87.50
Congdon et al.[35]	2020	Rwanda	Facility based	273	13.00	75.00
Olawoye et al.[36]	2011	Nigeria	Facility based	184	3.80	75.00
Oladigbolu et al.[37]	2014	Nigeria	Facility based	644	46.40	87.50
Bulus et al.[38]	2021	Nigeria	Facility based	116	43.40	87.50
Imam et al.[39]	2011	Nigeria	Facility based	288	29.00	75.00
Saa et al.[40]	2017	Togo	Facility based	1003	2.00	75.00
Semanyenzi et al.[41]	2015	Rwanda	Facility based	232	5.00	87.50
Ngonyani et al.[42]	2018	Tanzania	Facility based	370	13.00	75.00
Javaloy et al.[43]	2020	Cameroon	Facility based	263	2.60	75.00
Umerji et al[44]	2020	Zambia	Facility based	197	9.60	75.00
Moodley et al[45]	2019	South Africa	Facility based	128	9.00	87.50
Ahmad et al[46]	2011	Sudan	Facility based	145	4.20	75.00
Assoumou et al[47]	2024	Gabon	Facility based	240	15.30	87.50

Note: PPVO: Poor post-operative visual outcome.

### Meta-analysis on the pooled estimate of post-operative visual outcome of cataract surgery in Sub-Saharan Africa

**Fig 2:**
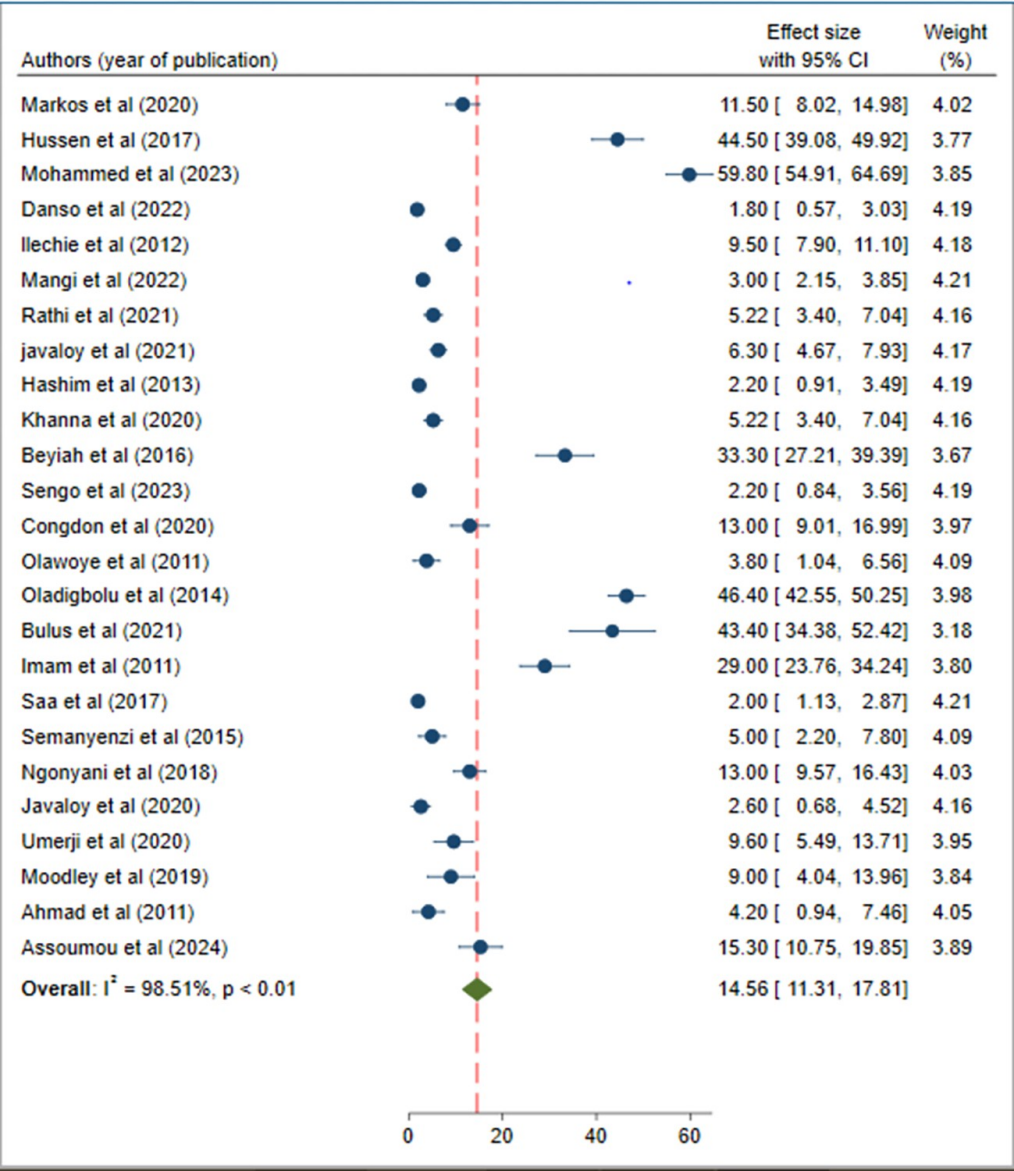
Forest plot of the pooled prevalence of poor post-operative visual outcome of cataract surgery in Sub-Saharan Africa.

The pooled prevalence of poor post-operative visual outcome of cataract surgery in SSA was 14.56% (95% CI 11.31, 17.81). As shown in the forest plot below, statistically significant heterogeneity was observed (I-squared = 98.51%; p<0.01). (Fig 2). Therefore, the pooled effect size was estimated using the random-effect model. In addition, the significant magnitude of heterogeneity also indicates the need to conduct subgroup analysis to identify the sources of heterogeneity across studies. In terms of individual prevalence, eastern Ghana and Ethiopia had the lowest (1.8%) and highest (59.8%) respectively (Fig 2).

### Subgroup analysis

**Fig 3:**
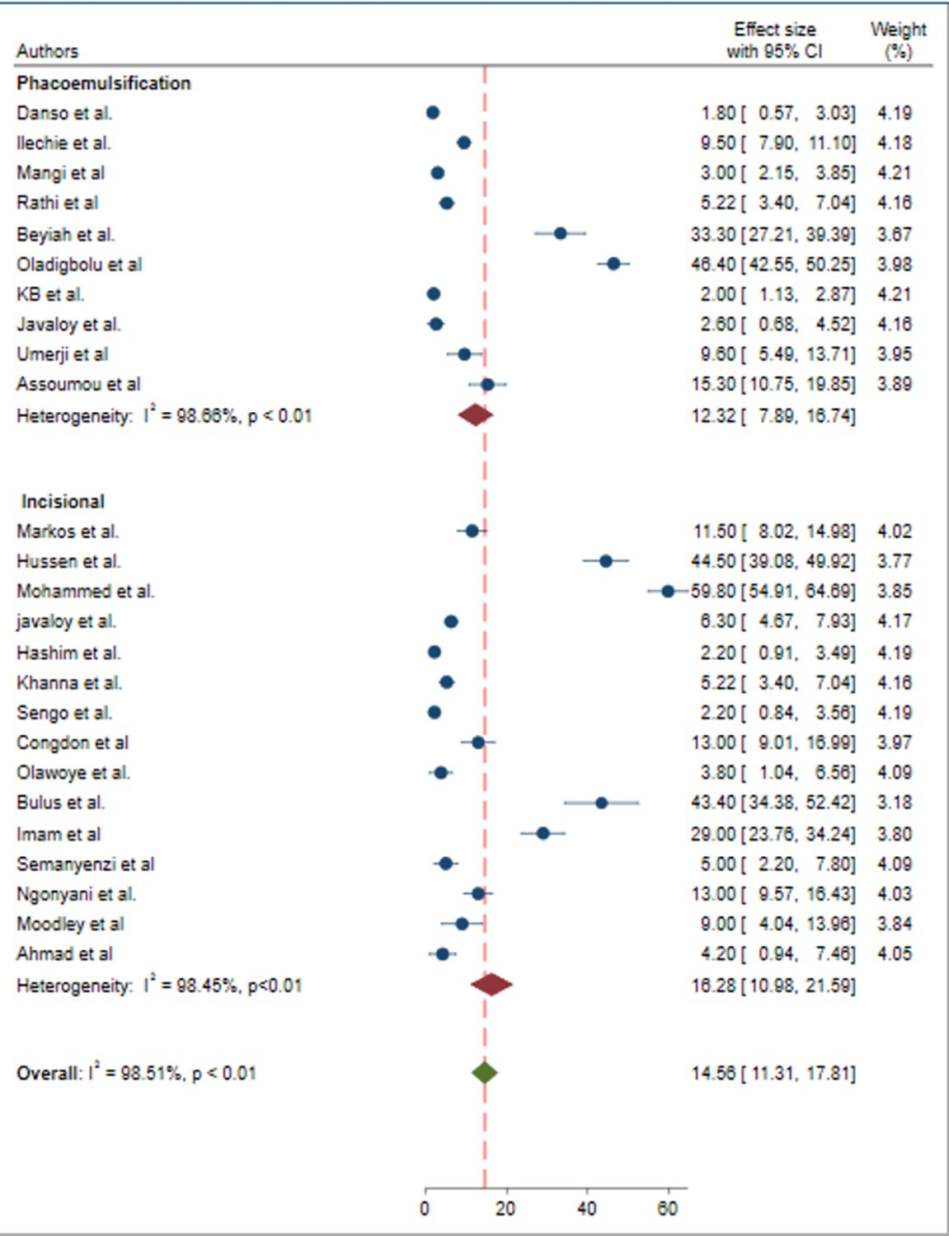
Forest plot of the pooled prevalence of poor post-operative visual outcome of cataract surgery in Sub-Saharan Africa by the type of cataract surgery.

**Fig 4:**
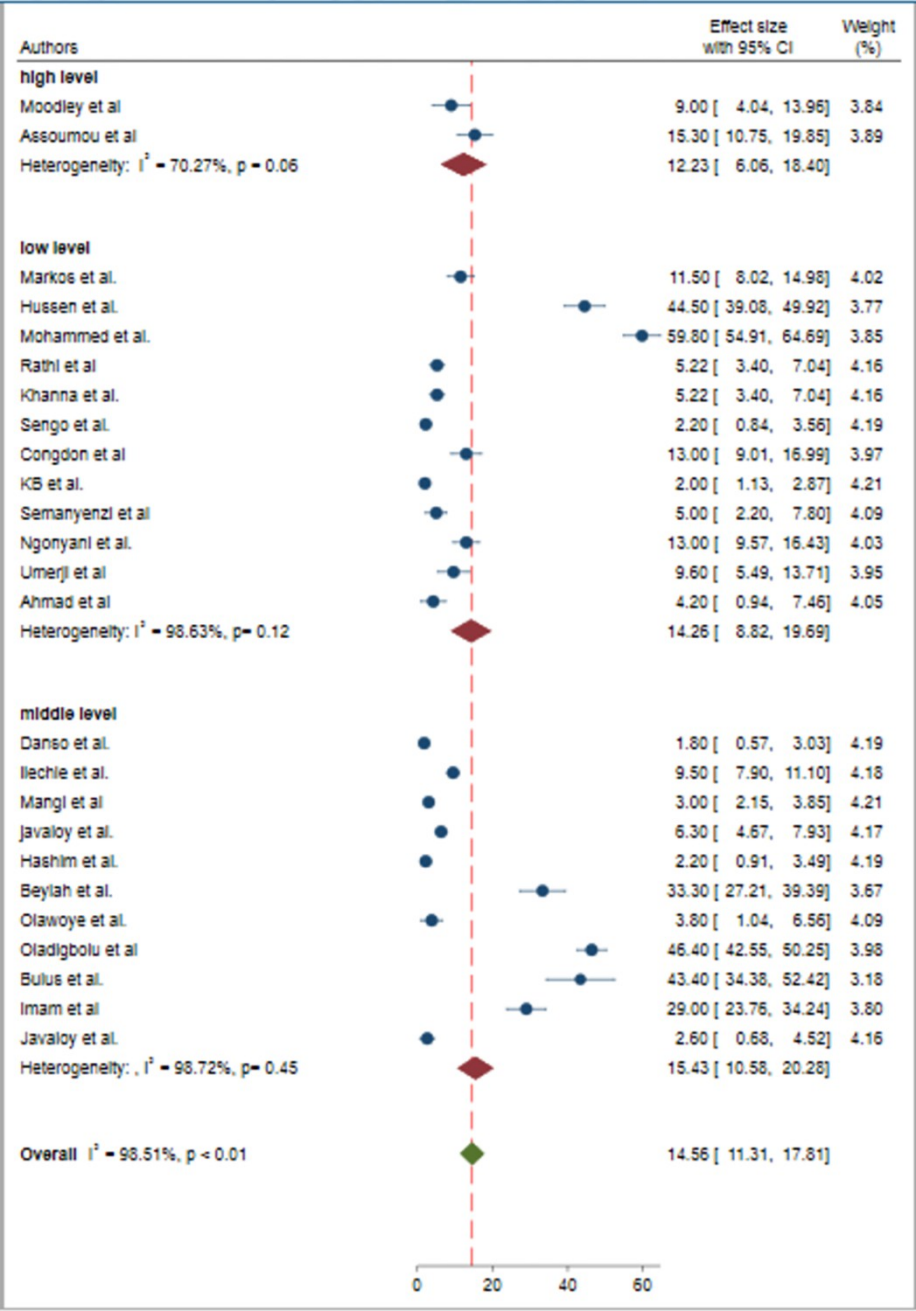
Forest plot of the pooled prevalence of poor post-operative visual outcome of cataract surgery in Sub-Saharan Africa by economic level of countries.

To assess the potential source of heterogeneity, subgroup analysis was performed based on the type of cataract surgery (incisional/phacoemulsification) (Fig 3) and the economic level of countries (low income/middle income/high income) (Fig 4). However, statistically significant heterogeneity was observed by only the type of cataract surgery (p<0.01). Therefore, subgroup analysis by the type of cataract surgery revealed the pooled prevalence of poor post-operative visual outcome was found lower in phacoemulsification, with a sub-pooled prevalence of 12.32% (95% CI 7.89, 16.74) compared to incisional cataract surgery, which had a sub-pooled prevalence of 16.28% (95% CI 10.98, 21.59).

### Publication bias

**Fig 5:**
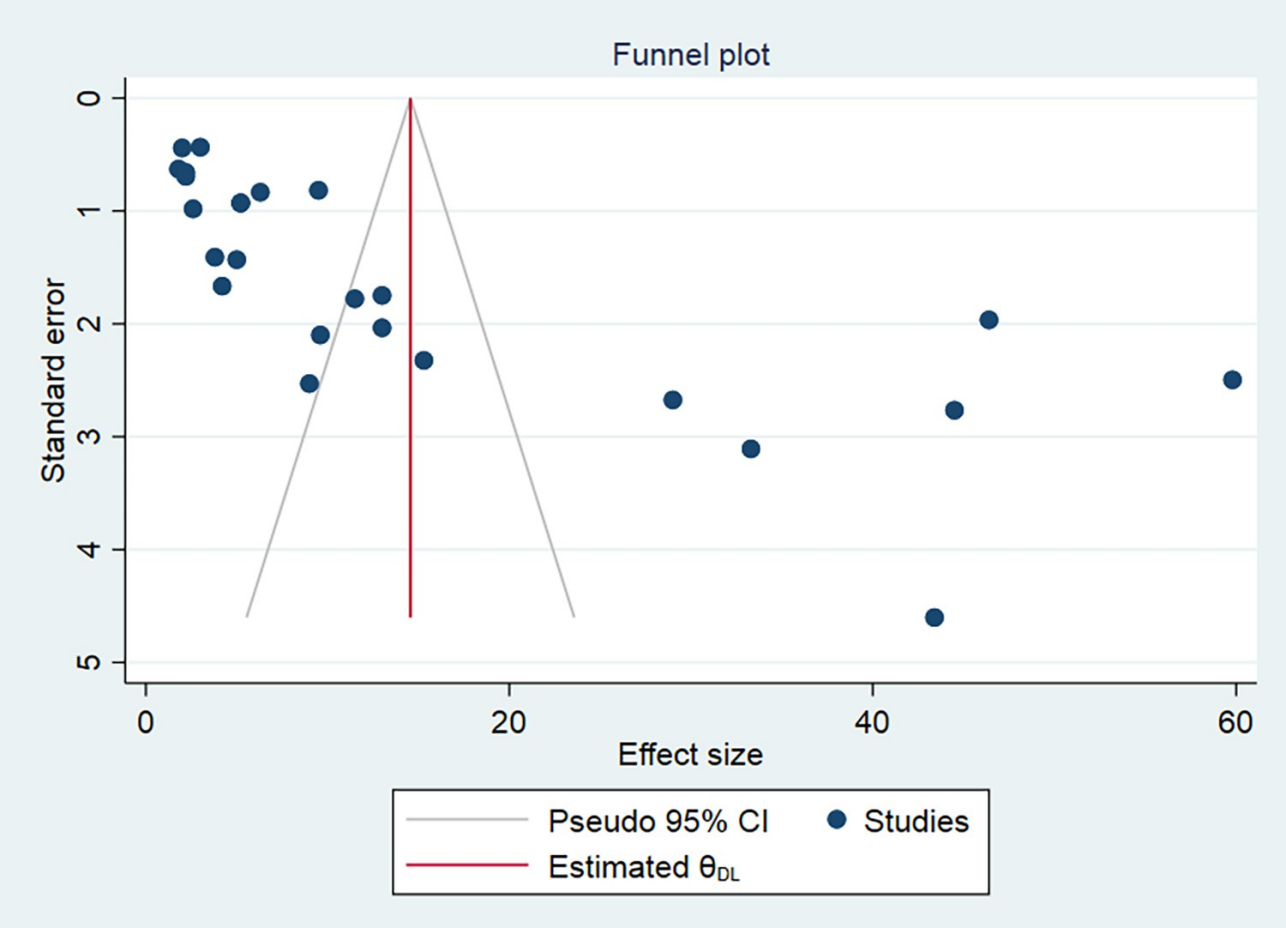
Funnel plot of the studies included in a systematic review and meta-analysis of the pooled magnitude of poor post-operative visual outcome of cataract surgery in sub-Saharan Africa.

**Fig 6:**
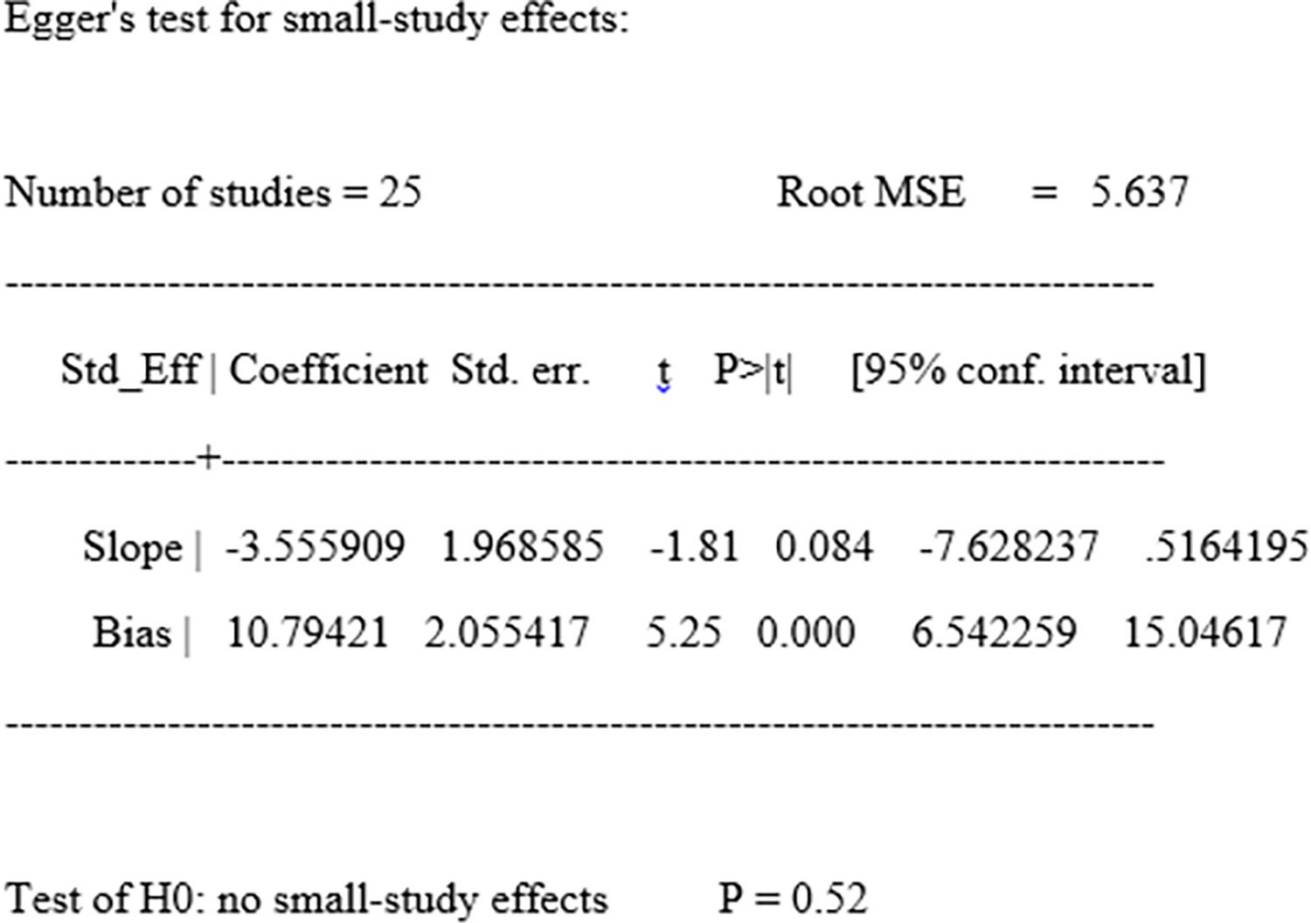
Egger’s test of the studies included in a systematic review and meta-analysis of the pooled magnitude of poor post-operative visual outcome of cataract surgery in sub-Saharan Africa.

The funnel plot was visually inspected to assess potential publication bias, which was statistically supported by Begg’s and Egger’s tests. The funnel plot indicated an asymmetrical distribution of publications in a large inverted funnel revealing the presence of publication bias (Fig 5). However, the statistical confirmations: The Begg and Egger tests revealed no publication bias among the studies included to estimate the pooled prevalence of poor postoperative visual outcome of cataract surgery, with p-values of (p = 0.68) and (p = 0.52), respectively (Fig 6).

### Associated factors of post-operative visual outcome of cataract surgery

**Fig 7:**
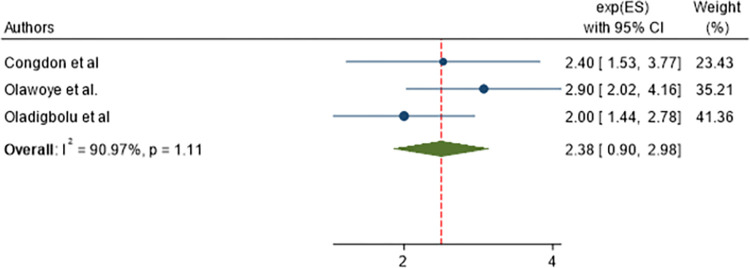
Forest plot showing pooled odds ratio (log scale) of the association between of age of participants and poor post-operative visual outcome.

**Fig 8:**
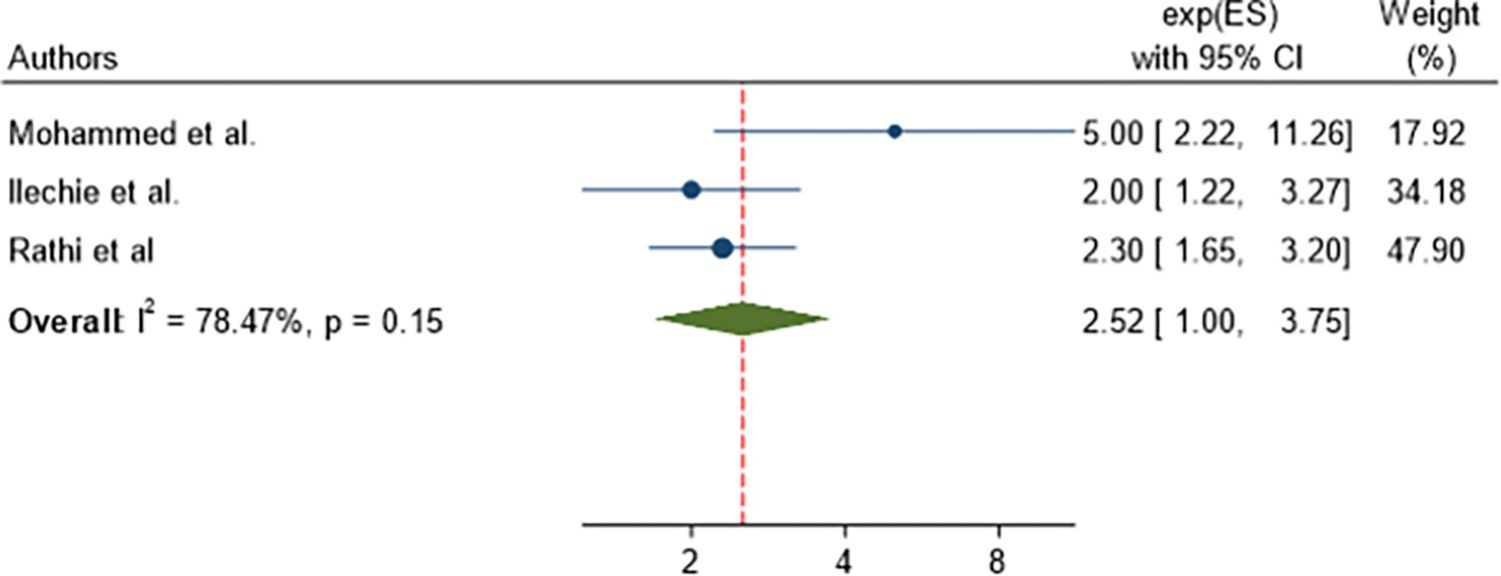
Forest plot showing pooled odds ratio (log scale) of the association between the presence of pre-operative astigmatism and poor post-operative visual outcome.

In this study, we assessed factors associated with poor post-operative visual outcome of cataract surgery. A separate analysis was conducted for each factor, which was cogitated in this meta-analysis. Variables assessed were: age of participants, presence of pre-operative astigmatism, presence of intra-operative complications, and presence of post-operative complications. From those variables; the Age of participants and the presence of pre-operative astigmatism were not significantly associated with poor post-operative visual outcome with p = 1.11 and 0.15 respectively as shown in Figs 7 and 8. However, the presence of intra-operative complications and postoperative complications were significantly associated with poor post-operative visual outcome.

**Fig 9:**
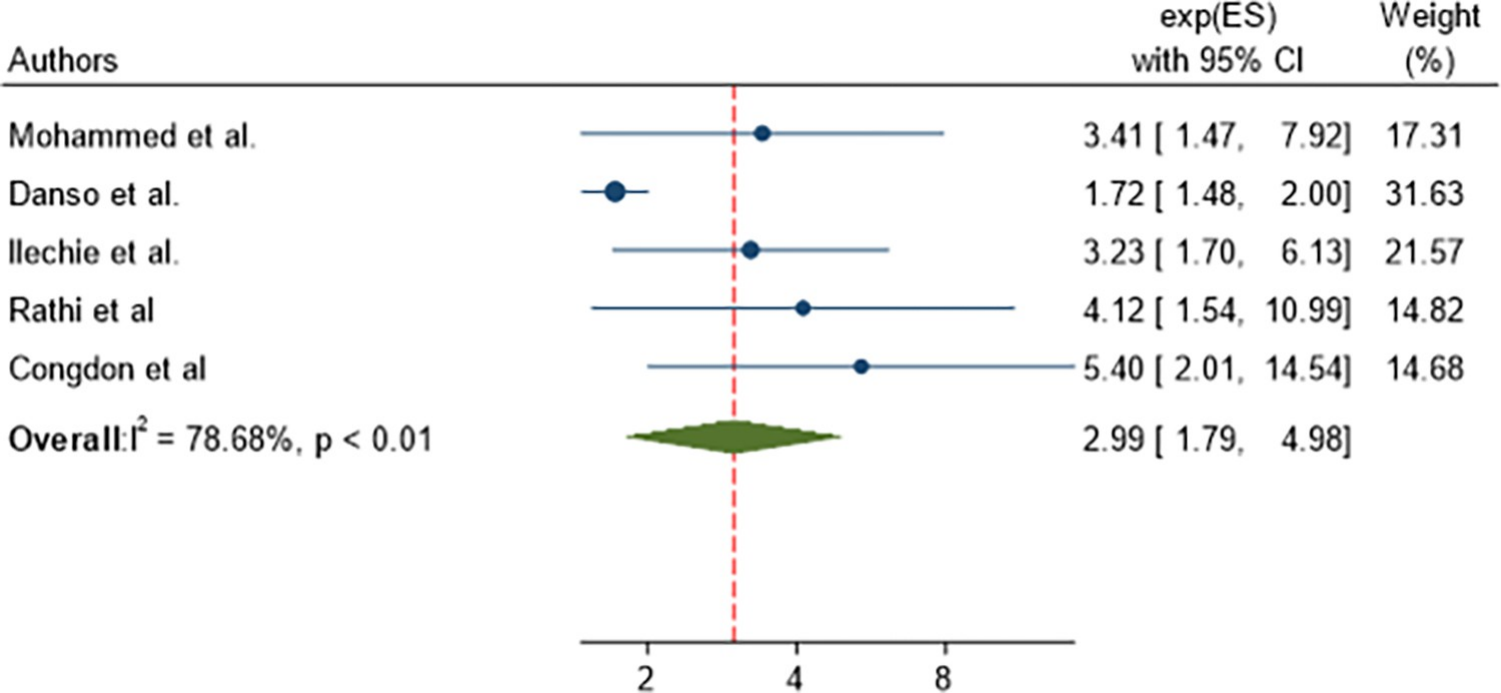
Forest plot showing pooled odds ratio (log scale) of the association between the presence of intra-operative complication and poor post-operative visual outcome.

Cataract-operated patients with intra-operative complications were 2.99 times (AOR = 2.99, 95% CI: 1.79, 4.98) more likely to have poor post-operative visual outcome than those with no intra-operative complications (Fig 9).

**Fig 10:**
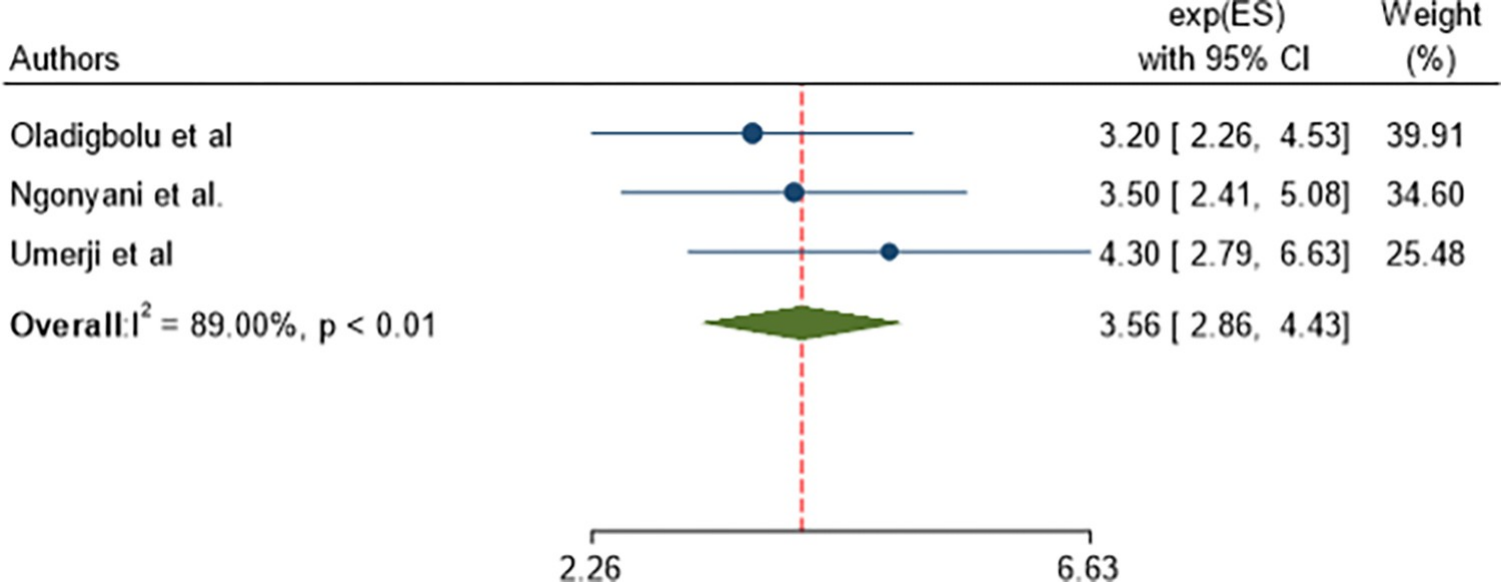
Forest plot showing pooled odds ratio (log scale) of the association between postoperative complication and poor post-operative visual outcome.

Cataract-operated patients with post-operative complications were 3.56 times (AOR = 3.56, 95% CI: 2.86, 4.43) more likely to have poor post-operative visual outcome than those with no post-operative complication (Fig 10).

## Discussion

This systematic review and meta-analysis aimed to estimate the pooled prevalence of poor post-operative visual outcome and associated factors of cataract surgery in sub-Saharan Africa. Accordingly, the pooled prevalence of poor post-operative visual outcomes of cataract surgery in sub-Saharan Africa was 14.56%. The result of this meta-analysis was consistent with the findings from Spain,15% [15], and India,16.6% [17].

However, it was higher than a study in the United States,4% [15], Nepal,9.5% [48], Asia,10.6% [49] and eastern China,11% [50]. This discrepancy might be due to the limited access to eye care service and surgical facilities in sub-Saharan Africa compared to regions such as Western India and certain parts of Asia, leading to delayed diagnosis, reduced surgical opportunities, and inadequate follow-up care after cataract surgery [51].

The current pooled prevalence of poor post-operative visual outcome was found lower than the studies reported from Libya,22.5% [52] and Algeria,29% [53]. This might be due to the difference in investment in training and resources as some countries within SSA such as Ghana and Kenya may have prioritized investment in training ophthalmic surgeons and eye care professionals, as well as providing essential resources and equipment for cataract surgeries [54]. This investment can lead to better surgical outcomes.

In the subgroup analysis, the pooled prevalence of poor post-operative visual outcome of cataract surgery was significantly varied by the type of cataract surgery. The pooled estimate of poor post-operative visual outcomes of cataract surgery was 12.32% in phacoemulsification and 16.28% in incisional surgeries. Based on this subgroup analysis the pooled estimate of poor post-operative visual outcome were significantly lower in phacoemulsification than incisional. The discrepancy in the type of cataract surgery might be due to, once the procedure is mastered the advanced technology and precision associated with phacoemulsification, which allows for smaller incisions, smaller changes in astigmatism, quicker recovery, and reduced complication rates [55]. In comparison, incisional surgeries, such as ECCE, ICCE, and SICS, may involve larger incisions, which can increase the risk of complications like astigmatism or infection potentially leading to poorer post-operative visual outcomes [56].

The odds of poor post-operative visual outcome were higher among operated patients with intra-operative complications than their counterparts. This result was supported by findings from Finland [57] and Spain [24], which similarly showed that those operated patients with intra-operative complications had higher odds of poor outcomes. The possible justification offered is that intra-operative complications (such as posterior capsule rupture, intra-operative hemorrhage, vitreous loss, zonular dehiscence, and retrobulbar hemorrhage, among others) during cataract surgery pose significant challenges including a higher risk of inflammation and compromised healing, risk in retinal and macular pathology, and the higher likelihood of refractive error.

The presence of postoperative complications (for instance, post-operative inflammations like uveitis, vitritis, endophthalmitis, and inflammatory cystoid macular edema, as well as other complications like posterior capsular opacifications among others)is another factor that is significantly associated with poor post-operative visual outcome in which, operated patients with postoperative complications had higher odds of poor post-operative visual outcomes as compared to their counterparts. This finding was supported by a study from Japan [58]. The reason might be due to the inflammatory response impairing the healing process, along with capsular fibrosis, both of which contribute to structural or functional damage to the eye and can negatively impact visual recovery [51].

This study highlights the critical need for improving access to advanced surgical techniques, such as phacoemulsification, in SSA to reduce the high rates of poor post-operative visual outcomes. Investing in specialized training and equipment for cataract surgeons should be a key policy focus to enhance surgical precision and reduce complications. Additionally, policies should emphasize consistent post-operative care, efficient management of inflammation and optimizing the use of neodymium-doped yttrium aluminum garnet (Nd) laser capsulotomy for better management of posterior capsular opacification (PCO), thereby mitigating long-term visual impairment after surgery. Future research should explore cost-effective strategies to introduce advanced technologies in resource-limited settings and identify best practices for reducing intra-operative complications. Furthermore, long-term studies are needed to assess the sustainability of surgical outcomes and guide future interventions.

### Strengths and limitations of the study

There were strengths and shortcomings in this study. To the best of our knowledge, this systematic review and meta-analysis is the first of its kind that was conducted to estimate the poor post-operative visual outcome and associated factors in sub-Saharan Africa. Moreover, the review’s overall quality was enhanced by a comprehensive literature search and JBI evaluation of the articles. Notably, our results emphasize the need for additional population-based studies on cataract surgery outcomes in this region, providing a basis for future research and policy development. This study, however, had limitations. Due to the absence of studies from all Sub-Saharan regions in this meta-analysis, the results might need to be interpreted carefully.

## Conclusion

This meta-analysis revealed that a substantial proportion of cataract-operated patients had poor postoperative visual outcomes. The presence of intra-operative complications and postoperative complications were independent predictors of poor postoperative visual outcome.

Therefore, improvement of postoperative visual outcomes through decreasing intra-operative complications and consistent post-operative care with efficient management of inflammation are pivotal and need significant emphasis. Additionally, investing in specialized training and equipment for ophthalmic surgeons should be a key policy focus to enhance surgical precision and reduce complications.

## Supporting information

S1 File PRISMA 2020 checklist. (PDF)

S2 File The minimal anonymized data set. (XLSX)

S1 Table All studies identified in the literature search (n = 201). (PDF)
