# Supplementary material for: Through the fog: Systematic review and meta-analysis of the prevalence and associated factors of poor post-operative visual outcome of cataract surgery in Sub-Saharan Africa
Source: PLoS One. 2024 Dec 9;19(12):e0315263. doi: 10.1371/journal.pone.0315263 (PMC11627423; doi:10.1371/journal.pone.0315263)
Supplement: S1 Table — (PDF) [file pone.0315263.s003.pdf]

S1 Table. All studies identified in the literature search (n=201).

No	Articles	Included/excluded
1	Limburg H, Foster A, Vaidyanathan K, Murthy GV. Monitoring visual outcome of cataract surgery in India. Bulletin of the World Health Organization. 1999;77(6):455.	Excluded
2	Limburg H, Foster A, Gilbert C, Johnson GJ, Kyndt M. Routine monitoring of visual outcome of cataract surgery. Part 1: Development of an instrument. British journal of ophthalmology. 2005 Jan 1;89(1):45-9.	Excluded
3	Limburg H. Monitoring cataract surgical outcomes: methods and tools. Community Eye Health. 2002;15(44):51.	Excluded
4	Thapa SS, Khanal S, Paudyal I, Twyana SN, Ruit S, van Rens GH. Outcomes of cataract surgery: a population-based developing world study in the Bhaktapur district, Nepal. Clinical & experimental ophthalmology. 2011 Dec;39(9):851-7.	Excluded
5	Markos CM, Tamrat LT, Asferaw MA. Outcomes and Associated Factors of Cataract Surgery Among Adults Attending a Tertiary Hospital in Addis Ababa, Ethiopia. Patient related outcome measures. 2020;11(34):231-9.	Included
6	Hussen MS, Gebreselassie KL, Seid MA, Belete GT. Visual outcome of cataract surgery at Gondar University Hospital Tertiary Eye Care and Training Center, North West Ethiopia. Clinical optometry. 2017;9:19-23.	Included
7	Hashmi FK, Khan QA, Chaudhry TA, Ahmad K. Visual outcome of cataract surgery. J Coll Physicians Surg Pak. 2013;23(6):448.	Excluded
8	Norregaard JC, Hindsberger C, Alonso J, Bellan L, Bernth-Petersen P, Black C, Dunn E, Andersen TF, Espallargues M, Anderson GF. Visual outcomes of cataract surgery in the United States, Canada, Denmark, and Spain: report from the international cataract surgery outcomes study. Archives of Ophthalmology. 1998 Aug 1;116(8):1095-100.	Excluded
9	Norregaard JC, Bernth-Petersen P, Alonso J, Andersen TF, Anderson GF. Visual functional outcomes of cataract surgery in the United States, Canada, Denmark, and Spain: report of the International Cataract Surgery Outcomes Study. Journal of Cataract & Refractive Surgery. 2003 Nov 1;29(11):2135-42.	Excluded
10	Noertjojo K, Mildon D, Rollins D, Law F, Blicker J, Courtright P, Sibley L, Bassett K. Cataract surgical outcome at the Vancouver Eye Care Centre: can it be predicted using current data?. Canadian journal of ophthalmology. 2004 Feb 1;39(1):38-47.	Excluded
11	Norregaard JC, Schein OD, Anderson GF, Alonso J, Dunn E, Black C, Andersen TF, Bernth-Petersen P, Bellan L, Espallargues	Excluded

	M. International variation in ophthalmologic management of patients with cataracts: results from the International Cataract Surgery Outcomes Study. Archives of ophthalmology. 1997 Mar 1;115(3):399-403.	
12	Lundström M, Goh PP, Henry Y, Salowi MA, Barry P, Manning S, Rosen P, Stenevi U. The changing pattern of cataract surgery indications: a 5-year study of 2 cataract surgery databases. Ophthalmology. 2015 Jan 1;122(1):31-8.	Excluded
13	Cook C. How to improve the outcome of cataract surgery. Community Eye Health. 2000;13(35):37.	Excluded
14	Wegener M, Alsbirk PH, Højgaard-Olsen K. Outcome of 1000 consecutive clinic-and hospital-based cataract surgeries in a Danish county. Journal of Cataract & Refractive Surgery. 1998 Aug 1;24(8):1152-60.	Excluded
15	Mohammed J, Assegid S, Fekadu L, Kabeta T. Cataract Surgery Visual Outcome and Associated Factors Among Adults Attended Jimma University Medical Center, Jimma, Southwest Ethiopia. Clinical Ophthalmology. 2023;12(45):3341-51.	Included
16	Danso-Appiah A, Mensah-Debrah A, Akuffo KO, Nortey P, Junior IOD. Postoperative vision outcomes after cataract surgery in the Eastern region of Ghana. Clinical Ophthalmology. 2022;32(2):320-45.	Included
17	Yorston D, Gichuhi S, Wood M, Foster A. Does prospective monitoring improve cataract surgery outcomes in Africa?. British Journal of Ophthalmology. 2002 May 1;86(5):543-7.	Excluded
18	Olawoye OO, Ashaye AO, Bekibele CO, Ajayi BG. Visual outcome after cataract surgery at the University College Hospital, Ibadan. Annals of Ibadan Postgraduate Medicine. 2011;9(1):8-13.	Excluded
19	Limburg H, Foster A, Gilbert C, Johnson GJ, Kyndt M, Myatt M. Routine monitoring of visual outcome of cataract surgery. Part 2: Results from eight study centres. British Journal of Ophthalmology. 2005 Jan 1;89(1):50-2.	Excluded
20	Yorston D, Wood M, Foster A. Results of cataract surgery in young children in east Africa. British journal of ophthalmology. 2001 Mar 1;85(3):267-71.	Excluded
21	Buchan JC, Dean WH, Foster A, Burton MJ. What are the priorities for improving cataract surgical outcomes in Africa? Results of a Delphi exercise. International ophthalmology. 2018 Aug;38:1409-14.	Excluded
22	Onakpoya OH, Bekibele CO, Adegbehingbe SA. Cataract surgical outcomes in diabetic patients: case control study. Middle East African journal of ophthalmology. 2009 Apr 1;16(2):88-91.	Excluded
23	Khandekar R, Sudhan A, Jain BK, Deshpande M, Dole K, Shah M, Shah S. Impact of cataract surgery in reducing visual	Excluded

	impairment: a review. Middle East African Journal of Ophthalmology. 2015 Jan 1;22(1):80-5.	
24	Gogate P, Parbhoo D, Ramson P, Budhoo R, Øverland L, Mkhize N, Naidoo K, Levine S, Du Bryn A, Benjamin L. Surgery for sight: outcomes of congenital and developmental cataracts operated in Durban, South Africa. Eye. 2016 Mar;30(3):406-12.	Excluded
25	Duke RE, Adio A, Oparah SK, Odey F, Eyo OA. Evaluation of a public child eye health tertiary facility for pediatric cataract in southern Nigeria I: Visual acuity outcome. The Open Ophthalmology Journal. 2016;10:119.	Excluded
26	Courtright P. Childhood cataract in sub-Saharan Africa. Saudi Journal of Ophthalmology. 2012 Jan 1;26(1):3-6.	Excluded
27	Limburg H, Foster A, Gilbert C, Johnson GJ, Kyndt M. Routine monitoring of visual outcome of cataract surgery. Part 1: Development of an instrument. British journal of ophthalmology. 2005 Jan 1;89(1):45-9.	Excluded
28	Malik AR, Qazi ZA, Gilbert C. Visual outcome after high volume cataract surgery in Pakistan. British journal of ophthalmology. 2003 Aug 1;87(8):937-40.	Excluded
29	Naeem M, Khan A, Khan MZ, Adil M, Abbas SH, Khan MU, Naz SM. Cataract: trends in surgical procedures and visual outcomes; a study in a tertiary care hospital. JPMA-Journal of the Pakistan Medical Association. 2012 Mar 1;62(3):209.	Excluded
30	Ilechie AA, Boadi-Kusi B, Ndudiri O, Ofori E. Evaluation of post-operative visual outcomes of cataract surgery in Ghana. International Journal of Health Research. 2012;5(1):35-42.	Included
31	Mangi M, Bashir MK, Inam M. Outcome of Cataract Surgery at Secondary Eye Care Facility in Karachi. Pakistan Journal of Ophthalmology. 2022;38(2):1245-56.	Included
32	Rathi VM, Khanna RC, Guizie E, Singh G, Nishant K, Sandhu S, et al. Cataract Surgery Visual Outcomes and Associated Risk Factors in Liberia. Investigative Ophthalmology & Visual Science. 2020;61(7):1675-80.	Included
33	Javaloy J, Moya T, Muñoz G, Albarrán-Diego C, Valls-Martínez J, Montalbán R, et al. Efficacy, safety and visual outcomes of cataract surgeries performed during blindness prevention programs in different locations in Kenya. Graefe's Archive for Clinical and Experimental Ophthalmology. 2021;259(2):1215-24.	Included
34	Limburg H, Foster A, Gilbert C, Johnson GJ, Kyndt M. Routine monitoring of visual outcome of cataract surgery. Part 1: Development of an instrument. British journal of ophthalmology. 2005 Jan 1;89(1):45-9.	Excluded

35	Malik AR, Qazi ZA, Gilbert C. Visual outcome after high volume cataract surgery in Pakistan. British journal of ophthalmology. 2003 Aug 1;87(8):937-40.	Excluded
36	Rabiu MM, Jenf M, Fituri S, Choudhury A, Agbabiaka I, Mousa A. Prevalence and causes of visual impairment and blindness, cataract surgical coverage and outcomes of cataract surgery in Libya. Ophthalmic epidemiology. 2013 Feb 1;20(1):26-32.	Excluded
37	Fortané M, Bensaid P, Resnikoff S, Seini K, Landreau N, Paugam JM, Nagot N, Mura T, Serrand C, Villain M, Daien V. Outcomes of cataract surgery performed by non-physician cataract surgeons in remote North Cameroon. British Journal of Ophthalmology. 2019 Aug 1;103(8):1042-7.	Excluded
38	Ezegwui IR, Ajewole J. Monitoring cataract surgical outcome in a Nigerian mission hospital. International ophthalmology. 2009 Feb;29:7-9.	Excluded
39	Briesen S, Roberts H, Lewallen S. The importance of biometry to cataract outcomes in a surgical unit in Africa. Ophthalmic Epidemiology. 2010 Aug 1;17(4):196-202.	Excluded
40	Bowman RJ, Kabiru J, Negretti G, Wood ML. Outcomes of bilateral cataract surgery in Tanzanian children. Ophthalmology. 2007 Dec 1;114(12):2287-92.	Excluded
41	Thanigasalam T, Reddy SC, Zaki RA. Factors associated with complications and postoperative visual outcomes of cataract surgery; a study of 1,632 cases. Journal of ophthalmic & vision research. 2015 Oct;10(4):375.	Excluded
42	Thanigasalam T, Reddy SC, Zaki RA. Factors associated with complications and postoperative visual outcomes of cataract surgery; a study of 1,632 cases. Journal of ophthalmic & vision research. 2015 Oct;10(4):375.	Excluded
43	Courtright P, Metcalfe N, Hoechsmann A, Chirambo M, Lewallen S, Barrows J, Witte C, Kanjaloti S, Mtambo O, Chipeta T, Mekisini G. Cataract surgical coverage and outcome of cataract surgery in a rural district in Malawi. Canadian journal of ophthalmology. 2004 Feb 1;39(1):25-30.	Excluded
44	Obiudu HC, Obi BI, Anyalebechi OC. Monitoring cataract surgical outcome in a public hospital in Orlu, South East Nigeria. Nigerian Medical Journal. 2009 Oct 1;50(4):77-9.	Excluded
45	Prasad M, Daigavane S, Kalode V. Visual outcome after cataract surgery in rural hospital of Wardha district: a prospective study. Journal of Clinical and Diagnostic Research. 2020 Feb 1;14(2):4-6.	Excluded
46	Mndeme FG, Mmbaga BT, Msina M, Mwende J, Vaitha SJ, Kim MJ, Macleod D, Burton MJ, Gilbert CE, Bowman R. Presentation, surgery and 1-year outcomes of childhood cataract surgery in Tanzania. British Journal of Ophthalmology. 2021 Mar 1;105(3):334-40.	Excluded

47	Hashmi FK, Khan QA, Chaudhry TA, Ahmad K. Visual outcome of cataract surgery. J Coll Physicians Surg Pak. 2013;23(6):448.	Included
48	Khanna RC, Rathi VM, Guizie E, Singh G, Nishant K, Sandhu S, et al. Factors associated with visual outcomes after cataract surgery: A cross-sectional or retrospective study in Liberia. PLoS One. 2020;15(5):e0233118.	Included
49	Beyiah KP. Outcome of Age Related Cataract Surgery at Mbingo Baptist Hospital Eye Unit, North West Region, Cameroon: University of Nairobi; 2016.	Included
50	Meltzer ME, Congdon N, Kymes SM, Yan X, Lansingh VC, Sisay A, Müller A, Chan VF, Jin L, Karumanchi SM, Guan C. Cost and expected visual effect of interventions to improve follow-up after cataract surgery: prospective review of early cataract outcomes and grading (PRECOG) study. JAMA ophthalmology. 2017 Feb 1;135(2):85-94.	Excluded
51	Mavrakanas N, Dhalla KA, Jecha J, Kapesa I, Odouard C, Murdoch I. Results and safety profile of trainee cataract surgeons in a community setting in East Africa. Indian journal of ophthalmology. 2016 Nov 1;64(11):818-21.	Excluded
52	Zitha AJ, Rampersad N. Cataract surgery outcomes: comparison of the extracapsular cataract extraction and manual small incision cataract surgery techniques. African Health Sciences. 2022 Apr 29;22(1):619-29.	Excluded
53	Khadem M. Outcomes of cataract surgery: implications for the developing world. Journal of medical systems. 1999 Aug;23:281-9.	Excluded
54	Rabiu MM, Kyari F, Ezelum C, Elhassan E, Sanda S, Murthy GV, Sivasubramaniam S, Glibert C. Review of the publications of the Nigeria national blindness survey: methodology, prevalence, causes of blindness and visual impairment and outcome of cataract surgery. Annals of African medicine. 2012 Jul 1;11(3):125-30.	Excluded
55	Oderinlo O, Hassan AO, Oluyadi FO, Ogunro AO, Okonkwo ON, Ulaikere MO, Ashano O. Refractive aim and visual outcome after phacoemulsification: A 2-year review from a Tertiary Private Eye Hospital in Sub-Saharan Africa. Nigerian journal of clinical practice. 2017;20(2):147-52.	Excluded
56	Sengo DB, Saravila PJ, Chivinde SS, Mate LM, Faquihe MF, Moragues R, et al. Assessment of cataract surgery outcomes in Nampula (Mozambique): visual acuity, visual function and quality of life. Graefe's Archive for Clinical and Experimental Ophthalmology. 2023;261(6):1597-608.	Included
57	Congdon N, Yan X, Lansingh V, Sisay A, Müller A, Chan V, et al. Assessment of cataract surgical outcomes in settings where follow-up is poor: PRECOG, a multicentre observational study. The Lancet Global health. 2013;1(1):e37-e45.	Included

58	Olawoye O, Ashaye A, Bekibele C, Ajayi B. Visual outcome after cataract surgery at the university college hospital, Ibadan. Annals of Ibadan Postgraduate Medicine. 2011;9(1):8-13.	Included
59	Oladigbolu KK, Rafindadi AL, Mahmud-Ajeigbe AF, Chinda D, Pam V, Samaila E. Outcome of cataract surgery in rural areas of Kaduna State, Nigeria. Annals of African medicine. 2014;13(1):25-9.	Included
60	Monsudi KF, Mahmoud AO, Adepoju FG, Ibrahim A. Impact of cataract surgery on visual function and quality of life in Birnin Kebbi, Nigeria. Br J Med Health Sci. 2012;1(3):80-99.	Excluded
61	Giles K, Christelle D, Yannick B, Fricke OH, Wiedemann P. Cataract surgery with intraocular lens implantation in children aged 5-15 in local anaesthesia: visual outcomes and complications. Pan African Medical Journal. 2016 Sep 19;24(1).	Excluded
62	Sherwin JC, Dean WH, Schaefers I, Courtright P, Metcalfe N. Outcomes of manual small-incision cataract surgery using standard 22 dioptre intraocular lenses at Nkhoma Eye Hospital, Malawi. International ophthalmology. 2012 Aug;32:341-7.	Excluded
63	Gogate P, Vakil V, Khandekar R, Deshpande M, Limburg H. Monitoring and modernization to improve visual outcomes of cataract surgery in a community eyecare center in western India. Journal of Cataract & Refractive Surgery. 2011 Feb 1;37(2):328-34.	Excluded
64	Kapoor H, Chatterjee A, Daniel R, Foster A. Evaluation of visual outcome of cataract surgery in an Indian eye camp. British journal of ophthalmology. 1999 Mar 1;83(3):343-6.	Excluded
65	Matta S, Park J, Palamaner Subash Shantha G, Khanna RC, Rao GN. Cataract surgery visual outcomes and associated risk factors in secondary level eye care centers of LV Prasad Eye Institute, India. PLoS One. 2016 Jan 7;11(1):e0144853.	Excluded
66	Bekibele CO. A comparative evaluation of outcome of cataract surgery at Ago-Iwoye, Ogun State. Nigerian Journal of Surgical Research. 2004;6(1-2).	Excluded
67	Ugalahi MO, Uchendu OC, Ugalahi LO. Preoperative visual acuity of cataract patients at a tertiary hospital in sub-Saharan Africa: a 10-year review. Therapeutic Advances in Ophthalmology. 2019 Nov;11:2515841419886451.	Excluded
68	Wong TY. Cataract surgery programmes in Africa. British journal of ophthalmology. 2005 Oct 1;89(10):1231-2.	Excluded
69	Chirambo MC. Country-wide monitoring of cataract surgical outcomes. Community Eye Health. 2002;15(44):58.	Excluded
70	Bulus SS, Bakut AS, Diyale PS, Mahmoud Z. Visual outcome of cataract surgery from a free outreach camp among rural areas of Southern-Kaduna, Nigeria. Global Journal of Cataract Surgery and Research in Ophthalmology. 2022;1(1):15-20.	Included
71	Imam AU, Gilbert CE, Sivasubramaniam S, Murthy GV, Maini R, Rabiu MM, et al. Outcome of cataract surgery in Nigeria: visual acuity, autorefraction, and optimal intraocular lens powers—results from the Nigeria national survey. Ophthalmology. 2011;118(4):719-24.	Included

72	Saa N. Manual small incision cataract surgery: Experience of a regional eye care service in Togo. Journal Francais D'ophtalmologie. 2018;41(3):255-61.	Included
73	Olusanya BA, Baiyeroju AM, Fajola AO. Visual recovery after cataract surgery in children. Nigerian Journal of Ophthalmology. 2006;14(2):46-51.	Excluded
74	Trivedy J. Outcomes of high volume cataract surgeries at a Lions Sight First Eye Hospital in Kenya. Nepalese Journal of Ophthalmology. 2011;3(1):31-8.	Excluded
75	Tomkins O, Ben-Zion I, Moore DB, Helveston EE. Outcomes of pediatric cataract surgery at a tertiary care center in rural southern Ethiopia. Archives of ophthalmology. 2011 Oct 10;129(10):1293-7.	Excluded
76	Semanyenzi S. Outcome after small incision cataract surgery (SICS) and phacoemulsification at Kigali University Teaching Hospital. Rwanda Medical Journal. 2015;72(4):12-6.	Included
77	Ngonyani CH. Visual Outcome and Causes of Poor Visual Outcome of Manual Small Incision Cataract Surgery in Adult Patients at Muhimbili National Hospital from May 2017 to January 2018.Muhimbili University of Health and Allied Sciences.2018.45(2):1733-45.	Included
77	Javaloy J, Signes-Soler I, Moya T, Litila S. Cataract surgery in surgical camps: outcomes in a rural area of Cameroon. International Ophthalmology. 2021;41:283-92.	Included
78	Umerji F. Factors associated with the outcome of cataract surgery at university teaching hospitals-eye hospital in Lusaka, Zambia: The University of Zambia; 2020.	Included
79	Foster A. Cataract and “Vision 2020—the right to sight” initiative. British Journal of Ophthalmology. 2001 Jun 1;85(6):635-7.	Excluded
80	Olawoye O, Ashaye A, Bekibele C, Ajuwon AJ. Quality-of-life and visual function after manual small incision cataract surgery in South Western Nigeria. West African journal of medicine. 2012;31(2):114-9.	Excluded
81	Lewallen S, Williams TD, Dray A, Stock BC, Mathenge W, Oye J, Nkurikiye J, Kimani K, Müller A, Courtright P. Estimating incidence of vision-reducing cataract in Africa: a new model with implications for program targets. Archives of ophthalmology. 2010 Dec 1;128(12):1584-9.	Excluded
82	Ejimadu CS, Pedro-Egbe CN. Audit of visual outcome of cataract surgeries in a private eye hospital in Port Harcourt, Nigeria. Nigerian Journal of Ophthalmology. 2014;22(1):7-10.	Excluded
83	Moodley S, Alberto K. Visual outcomes in manual small incision cataract surgery versus phacoemulsification: a prospective comparative data analysis. South African Ophthalmology Journal. 2019;14(3):21-4.	Included

84	Ahmad S. Visual outcome of cataract surgery in a field hospital of sub-Saharan Africa. Pakistan Armed Forces Medical Journal. 2011;61(2):241-5.	Included
85	Assoumou P, DA MA, Maloumbi G, Matsanga O. Functional Results and Patient Satisfaction after Cataract Surgery by Phacoemulsification in Gabon: Résultats Fonctionnels et Satisfaction des Patients après Chirurgie de la Cataracte par Phacoémulsification au Gabon. HEALTH RESEARCH IN AFRICA. 2024;2(5).	Included
86	Mönestam E, Lundqvist B. Long-term visual outcome after cataract surgery: comparison of healthy eyes and eyes with age-related macular degeneration. Journal of Cataract & Refractive Surgery. 2012 Mar 1;38(3):409-14.	Excluded
87	Ondrácek O, Lokaj M. Visual outcome after congenital cataract surgery. Long-term clinical results. Scripta Med (Brno). 2003 Apr;76(2):95-102.	Excluded
88	Ondrácek O, Lokaj M. Visual outcome after congenital cataract surgery. Long-term clinical results. Scripta Med (Brno). 2003 Apr;76(2):95-102.	Excluded
89	Mollazadegan K, Lundström M. A study of the correlation between patient-reported outcomes and clinical outcomes after cataract surgery in ophthalmic clinics. Acta ophthalmologica. 2015 May;93(3):293-8.	Excluded
90	Mollazadegan K, Lundström M. A study of the correlation between patient-reported outcomes and clinical outcomes after cataract surgery in ophthalmic clinics. Acta ophthalmologica. 2015 May;93(3):293-8.	Excluded
91	Blomquist PH, Rugwani RM. Visual outcomes after vitreous loss during cataract surgery performed by residents. Journal of Cataract & Refractive Surgery. 2002 May 1;28(5):847-52.	Excluded
92	Ionides A, Minassian D, Tuft S. Visual outcome following posterior capsule rupture during cataract surgery. British journal of ophthalmology. 2001 Feb 1;85(2):222-4.	Excluded
93	Lindfield R, Vishwanath K, Ngounou F, Khanna RC. The challenges in improving outcome of cataract surgery in low and middle income countries. Indian Journal of Ophthalmology. 2012 Sep 1;60(5):464-9.	Excluded
94	Gogate P, Vakil V, Khandekar R, Deshpande M, Limburg H. Monitoring and modernization to improve visual outcomes of cataract surgery in a community eyecare center in western India. Journal of Cataract & Refractive Surgery. 2011 Feb 1;37(2):328-34.	Excluded
95	Yuan J, Wang X, Yang LQ, Xing YQ, Yang YN. Assessment of visual outcomes of cataract surgery in Tujia nationality in	Excluded

	Xianfeng County, China. International Journal of Ophthalmology. 2015;8(2):292.	
96	Lundstrom M, Behndig A, Kugelberg M, Montan P, Stenevi U, Pesudovs K. The outcome of cataract surgery measured with the Catquest-9SF. Acta ophthalmologica. 2011 Dec;89(8):718-23.	Excluded
97	Vijaya L, George R, Rashima A, Raju P, Arvind H, Baskaran M, Ramesh SV. Outcomes of cataract surgery in a rural and urban south Indian population. Indian journal of ophthalmology. 2010 May 1;58(3):223-8.	Excluded
98	Yorston D, Gichuhi S, Wood M, Foster A. Does prospective monitoring improve cataract surgery outcomes in Africa?. British Journal of Ophthalmology. 2002 May 1;86(5):543-7.	Excluded
99	Visser N, Nuijts RM, de Vries NE, Bauer NJ. Visual outcomes and patient satisfaction after cataract surgery with toric multifocal intraocular lens implantation. Journal of Cataract & Refractive Surgery. 2011 Nov 1;37(11):2034-42.	Excluded
100	Lavanya R, Wong TY, Aung T, Tan DT, Saw SM, Tay WT, Wang JJ. Prevalence of cataract surgery and post-surgical visual outcomes in an urban Asian population: the Singapore Malay Eye Study. British journal of ophthalmology. 2009 Mar 1;93(3):299-304.	Excluded
101	Thapa SS, Khanal S, Paudyal I, Twyana SN, Ruit S, van Rens GH. Outcomes of cataract surgery: a population-based developing world study in the Bhaktapur district, Nepal. Clinical & experimental ophthalmology. 2011 Dec;39(9):851-7.	Excluded
102	Vijayalakshmi P, Srivastava KK, Poornima B, Nirmalan P. Visual outcome of cataract surgery in children with congenital rubella syndrome. Journal of American Association for Pediatric Ophthalmology and Strabismus. 2003 Apr 1;7(2):91-5.	Excluded
103	Thevi T, Godinho MA. Predictive factors of visual outcome of Malaysian cataract patients: a retrospective study. International journal of ophthalmology. 2017;10(9):1452.	Excluded
104	Huang W, Zheng Y, Wang L, Huang S, Liu B, Jin L, Congdon NG, He M. Five-year incidence and postoperative visual outcome of cataract surgery in urban southern China: the Liwan Eye Study. Investigative ophthalmology & visual science. 2012 Dec 1;53(13):7936-42.	Excluded
105	Huang W, Zheng Y, Wang L, Huang S, Liu B, Jin L, Congdon NG, He M. Five-year incidence and postoperative visual outcome of cataract surgery in urban southern China: the Liwan Eye Study. Investigative ophthalmology & visual science. 2012 Dec 1;53(13):7936-42.	Excluded
106	Bourne RR, Dineen BP, Ali SM, Huq DN, Johnson GJ. Outcomes of cataract surgery in Bangladesh: results from a population based nationwide survey. British journal of ophthalmology. 2003 Jul 1;87(7):813-9.	Excluded

107	Lundström M, Goh PP, Henry Y, Salowi MA, Barry P, Manning S, Rosen P, Stenevi U. The changing pattern of cataract surgery indications: a 5-year study of 2 cataract surgery databases. Ophthalmology. 2015 Jan 1;122(1):31-8.	Excluded
108	Del Risco NE, Talbot CL, Moin KA, Manion GN, Brown AH, Walker SM, Zhong PS, Zhang H, Hoopes PC, Moshirfar M. Visual outcomes of cataract surgery in patients with previous history of implantable Collamer lens. Journal of Clinical Medicine. 2024 Jul 23;13(15):4292.	Excluded
109	Chan E, Mahroo OA, Spalton DJ. Complications of cataract surgery. Clinical and Experimental Optometry. 2010 Nov 1;93(6):379-89.	Excluded
110	Pager CK. Expectations and outcomes in cataract surgery: a prospective test of 2 models of satisfaction. Archives of ophthalmology. 2004 Dec 1;122(12):1788-92.	Excluded
111	Manning S, Barry P, Henry Y, Rosen P, Stenevi U, Lundström M. Cataract surgery outcomes in corneal refractive surgery eyes: Study from the European Registry of Quality Outcomes for Cataract and Refractive Surgery. Journal of Cataract & Refractive Surgery. 2015 Nov 1;41(11):2358-65.	Excluded
112	Das S, Khanna R, Mohiuddin SM, Ramamurthy B. Surgical and visual outcomes for posterior polar cataract. British Journal of Ophthalmology. 2008 Nov 1;92(11):1476-8.	Excluded
113	Jackson H, Garway-Heath D, Rosen P, Bird AC, Tuft SJ. Outcome of cataract surgery in patients with retinitis pigmentosa. British Journal of Ophthalmology. 2001 Aug 1;85(8):936-8.	Excluded
114	Jackson H, Garway-Heath D, Rosen P, Bird AC, Tuft SJ. Outcome of cataract surgery in patients with retinitis pigmentosa. British Journal of Ophthalmology. 2001 Aug 1;85(8):936-8.	Excluded
115	González N, Quintana JM, Bilbao A, Vidal S, de Larrea NF, Díaz V, Gracia J, IRYSS-Cataract Group. Factors affecting cataract surgery complications and their effect on the postoperative outcome. Canadian Journal of Ophthalmology. 2014 Feb 1;49(1):72-9.	Excluded
116	Ewe SY, Abell RG, Oakley CL, Lim CH, Allen PL, McPherson ZE, Rao A, Davies PE, Vote BJ. A comparative cohort study of visual outcomes in femtosecond laser-assisted versus phacoemulsification cataract surgery. Ophthalmology. 2016 Jan 1;123(1):178-82.	Excluded
117	Memon MN, Narsani AK, Nizamani NB. Visual outcome of unilateral traumatic cataract. J Coll Physicians Surg Pak. 2012 Aug 1;22(8):497-500.	Excluded
118	Limburg H, Foster A, Vaidyanathan K, Murthy GV. Monitoring visual outcome of cataract surgery in India. Bulletin of the World Health Organization. 1999;77(6):455.	Excluded
119	Limburg H, Foster A, Gilbert C, Johnson GJ, Kyndt M. Routine monitoring of visual outcome of cataract surgery. Part 1:	Excluded

	Development of an instrument. British journal of ophthalmology. 2005 Jan 1;89(1):45-9.	
120	Thapa SS, Khanal S, Paudyal I, Twyana SN, Ruit S, van Rens GH. Outcomes of cataract surgery: a population-based developing world study in the Bhaktapur district, Nepal. Clinical & experimental ophthalmology. 2011 Dec;39(9):851-7.	Excluded
121	Markos CM, Tamrat LT, Asferaw MA. Outcomes and Associated Factors of Cataract Surgery Among Adults Attending a Tertiary Hospital in Addis Ababa, Ethiopia. Patient related outcome measures. 2020;11(34):231-9.	Excluded
122	Hussen MS, Gebreselassie KL, Seid MA, Belete GT. Visual outcome of cataract surgery at Gondar University Hospital Tertiary Eye Care and Training Center, North West Ethiopia. Clinical optometry. 2017;9:19-23.	Excluded
123	Hashmi FK, Khan QA, Chaudhry TA, Ahmad K. Visual outcome of cataract surgery. J Coll Physicians Surg Pak. 2013;23(6):448.	Excluded
124	Norregaard JC, Hindsberger C, Alonso J, Bellan L, Bernth-Petersen P, Black C, Dunn E, Andersen TF, Espallargues M, Anderson GF. Visual outcomes of cataract surgery in the United States, Canada, Denmark, and Spain: report from the international cataract surgery outcomes study. Archives of Ophthalmology. 1998 Aug 1;116(8):1095-100.	Excluded
125	Bernth-Petersen P, Alonso J, Andersen TF, Anderson GF. Visual functional outcomes of cataract surgery in the United States, Canada, Denmark, and Spain: report of the International Cataract Surgery Outcomes Study. Journal of Cataract & Refractive Surgery. 2003 Nov 1;29(11):2135-42.	Excluded
126	Law F, Blicker J, Courtright P, Sibley L, Bassett K. Cataract surgical outcome at the Vancouver Eye Care Centre: can it be predicted using current data?. Canadian journal of ophthalmology. 2004 Feb 1;39(1):38-47.	Excluded
127	Lundström M, Goh PP, Henry Y, Salowi MA, Barry P, Manning S, Rosen P, Stenevi U. The changing pattern of cataract surgery indications: a 5-year study of 2 cataract surgery databases. Ophthalmology. 2015 Jan 1;122(1):31-8.	Excluded
128	Cook C. How to improve the outcome of cataract surgery. Community Eye Health. 2000;13(35):37.	Excluded
129	Outcome of 1000 consecutive clinic-and hospital-based cataract surgeries in a Danish county. Journal of Cataract & Refractive Surgery. 1998 Aug 1;24(8):1152-60.	Excluded
130	Mönestam E, Lundqvist B. Long-term visual outcome after cataract surgery: comparison of healthy eyes and eyes with age-related macular degeneration. Journal of Cataract & Refractive Surgery. 2012 Mar 1;38(3):409-14.	Excluded

131	Ondrácek O, Lokaj M. Visual outcome after congenital cataract surgery. Long-term clinical results. Scripta Med (Brno). 2003 Apr;76(2):95-102	Excluded
132	Visual outcome after congenital cataract surgery. Long-term clinical results. Scripta Med (Brno). 2003 Apr;76(2):95-102.	Excluded
133	Blomquist PH, Rugwani RM. Visual outcomes after vitreous loss during cataract surgery performed by residents. Journal of Cataract & Refractive Surgery. 2002 May 1;28(5):847-52.	Excluded
134	Ionides A, Minassian D, Tuft S. Visual outcome following posterior capsule rupture during cataract surgery. British journal of ophthalmology. 2001 Feb 1;85(2):222-4.	Excluded
135	Lindfield R, Vishwanath K, Ngounou F, Khanna RC. The challenges in improving outcome of cataract surgery in low and middle income countries. Indian Journal of Ophthalmology. 2012 Sep 1;60(5):464-9.	Excluded
136	Vakil V, Khandekar R, Deshpande M, Limburg H. Monitoring and modernization to improve visual outcomes of cataract surgery in a community eyecare center in western India. Journal of Cataract & Refractive Surgery. 2011 Feb 1;37(2):328-34.	Excluded
137	Vijaya L, George R, Rashima A, Raju P, Arvind H, Baskaran M, Ramesh SV. Outcomes of cataract surgery in a rural and urban south Indian population. Indian journal of ophthalmology. 2010 May 1;58(3):223-8.	Excluded
138	Yorston D, Gichuhi S, Wood M, Foster A. Does prospective monitoring improve cataract surgery outcomes in Africa?. British Journal of Ophthalmology. 2002 May 1;86(5):543-7.	Excluded
139	Visser N, Nuijts RM, de Vries NE, Bauer NJ. Visual outcomes and patient satisfaction after cataract surgery with toric multifocal intraocular lens implantation. Journal of Cataract & Refractive Surgery. 2011 Nov 1;37(11):2034-42.	Excluded
140	Lavanya R, Wong TY, Aung T, Tan DT, Saw SM, Tay WT, Wang JJ. Prevalence of cataract surgery and post-surgical visual outcomes in an urban Asian population: the Singapore Malay Eye Study. British journal of ophthalmology. 2009 Mar 1;93(3):299-304.	Excluded
141	Thapa SS, Khanal S, Paudyal I, Twyana SN, Ruit S, van Rens GH. Outcomes of cataract surgery: a population-based developing world study in the Bhaktapur district, Nepal. Clinical & experimental ophthalmology. 2011 Dec;39(9):851-7.	Excluded
141	Srivastava KK, Poornima B, Nirmalan P. Visual outcome of cataract surgery in children with congenital rubella syndrome. Journal of American Association for Pediatric Ophthalmology and Strabismus. 2003 Apr 1;7(2):91-5.	Excluded
142	Gichuhi S, Wood M, Foster A. Does prospective monitoring improve cataract surgery outcomes in Africa?. British Journal of Ophthalmology. 2002 May 1;86(5):543-7	Excluded

143	Wong TY, Aung T, Tan DT, Saw SM, Tay WT, Wang JJ. Prevalence of cataract surgery and post-surgical visual outcomes in an urban Asian population: the Singapore Malay Eye Study. British journal of ophthalmology. 2009 Mar 1;93(3):299-304.	Excluded
144	Khanal S, Paudyal I, Twyana SN, Ruit S, van Rens GH. Outcomes of cataract surgery: a population-based developing world study in the Bhaktapur district, Nepal. Clinical & experimental ophthalmology. 2011 Dec;39(9):851-7.	Excluded
145	Predictive factors of visual outcome of Malaysian cataract patients: a retrospective study. International journal of ophthalmology. 2017;10(9):1452	Excluded
146	Trivedy J. Outcomes of high volume cataract surgeries at a Lions Sight First Eye Hospital in Kenya. Nepalese Journal of Ophthalmology. 2011;3(1):31-8.	Excluded
147	Olusanya BA, Baiyeroju AM, Fajola AO. Visual recovery after cataract surgery in children. Nigerian Journal of Ophthalmology. 2006;14(2):46-51.	Excluded
148	Foster A. Cataract and "Vision 2020—the right to sight" initiative. British Journal of Ophthalmology. 2001 Jun 1;85(6):635-7.	Excluded
149	Pedro-Egbe CN. Audit of visual outcome of cataract surgeries in a private eye hospital in Port Harcourt, Nigeria. Nigerian Journal of Ophthalmology. 2014;22(1):7-10.	Excluded
150	Sherwin JC, Dean WH, Schaefers I, Courtright P, Metcalfe N. Outcomes of manual small-incision cataract surgery using standard 22 dioptre intraocular lenses at Nkhoma Eye Hospital, Malawi. International ophthalmology. 2012 Aug;32:341-7.	Excluded
151	Matta S, Park J, Palamaner Subash Shantha G, Khanna RC, Rao GN. Cataract surgery visual outcomes and associated risk factors in secondary level eye care centers of LV Prasad Eye Institute, India. PLoS One. 2016 Jan 7;11(1):e0144853.	Excluded
152	Pai SG, Kamath SJ, Kedia V, Shruthi K, Pai A. Cataract surgery in camp patients: a study on visual outcomes. Nepalese Journal of Ophthalmology. 2011;3(2):159-64.	Excluded
153	Bourne R, Dineen B, Jadoon Z, Lee PS, Khan A, Johnson GJ, Foster A, Khan D. Outcomes of cataract surgery in Pakistan: results from the Pakistan national blindness and visual impairment survey. British journal of ophthalmology. 2007 Apr 1;91(4):420-6.	Excluded
154	Onakpoya OH, Bekibele CO, Adegbehingbe SA. Cataract surgical outcomes in diabetic patients: case control study. Middle East African journal of ophthalmology. 2009 Apr 1;16(2):88-91.	Excluded
155	Cox JT, Subburaman GB, Munoz B, Friedman DS, Ravindran RD. Visual acuity outcomes after cataract surgery: high-volume versus low-volume surgeons. Ophthalmology. 2019 Nov 1;126(11):1480-9.	Excluded
156	Alió JL, Montalbán R, Peña-García P, Soria FA, Vega-Estrada A. Visual outcomes of a trifocal aspheric diffractive intraocular lens	Excluded

	with microincision cataract surgery. Journal of refractive surgery. 2013 Nov 1;29(11):756-61.	
157	Stifter E, Sacu S, Weghaupt H, König F, Richter-Müksch S, Thaler A, Velikay-Parel M, Radner W. Reading performance depending on the type of cataract and its predictability on the visual outcome. Journal of Cataract & Refractive Surgery. 2004 Jun 1;30(6):1259-67.	Excluded
158	Stifter E, Sacu S, Weghaupt H, König F, Richter-Müksch S, Thaler A, Velikay-Parel M, Radner W. Reading performance depending on the type of cataract and its predictability on the visual outcome. Journal of Cataract & Refractive Surgery. 2004 Jun 1;30(6):1259-67.	Excluded
159	Keel S, Xie J, Foreman J, Taylor HR, Dirani M. Population-based assessment of visual acuity outcomes following cataract surgery in Australia: the National Eye Health Survey. British Journal of Ophthalmology. 2018 Oct 1;102(10):1419-24.	Excluded
160	Tsai CY, Chang TJ, Kuo LL, Chou P, Woung LC. Visual outcomes and associated risk factors of cataract surgeries in highly myopic Taiwanese. Ophthalmologica. 2008 Feb 22;222(2):130-5.	Excluded
161	Altan T, Acar N, Kapran Z, Unver YB, Yurttaser S, KÜÇÜKSÜMER Y, Eser I. Acute-onset endophthalmitis after cataract surgery: success of initial therapy, visual outcomes, and related factors. Retina. 2009 May 1;29(5):606-12.	Excluded
162	Lundström M, Dickman M, Henry Y, Manning S, Rosen P, Tassignon MJ, Young D, Stenevi U. Risk factors for refractive error after cataract surgery: analysis of 282 811 cataract extractions reported to the European Registry of Quality Outcomes for cataract and refractive surgery. Journal of Cataract & Refractive Surgery. 2018 Apr 1;44(4):447-52.	Excluded
163	Lundström M, Dickman M, Henry Y, Manning S, Rosen P, Tassignon MJ, Young D, Stenevi U. Risk factors for refractive error after cataract surgery: analysis of 282 811 cataract extractions reported to the European Registry of Quality Outcomes for cataract and refractive surgery. Journal of Cataract & Refractive Surgery. 2018 Apr 1;44(4):447-52.	Excluded
164	Chirambo MC. Country-wide monitoring of cataract surgical outcomes. Community Eye Health. 2002;15(44):58.	Excluded
165	Carrim ZI, Richardson J, Wykes WN. Incidence and visual outcome of acute endophthalmitis after cataract surgery—the experience of an eye department in Scotland. British journal of ophthalmology. 2009 Jun 1;93(6):721-5.	Excluded
166	Yosar JC, Zagora SL, Grigg JR. Cataract surgery in short eyes, including nanophthalmos: visual outcomes, complications and refractive results. Clinical Ophthalmology. 2021 Nov 27:4543-51.	Excluded
167	Mozaffarieh M, Heinzl H, Sacu S, Wedrich A. Clinical outcomes of phacoemulsification cataract surgery in diabetes patients: visual	Excluded

	function (VF-14), visual acuity and patient satisfaction. Acta Ophthalmologica Scandinavica. 2005 Apr;83(2):176-83.	
168	Huang W, Huang G, Wang D, Yin Q, Foster PJ, He M. Outcomes of cataract surgery in urban southern China: the Liwan Eye Study. Investigative ophthalmology & visual science. 2011 Jan 1;52(1):16-20.	Excluded
169	Marcos S, Martinez-Enriquez E, Vinas M, de Castro A, Dorronsoro C, Bang SP, Yoon G, Artal P. Simulating outcomes of cataract surgery: important advances in ophthalmology. Annual review of biomedical engineering. 2021 Jul 13;23(1):277-306.	Excluded
170	Khan MT, Jan S, Hussain Z, Karim S, Khalid MK, Mohammad L. Visual outcome and complications of manual sutureless small incision cataract surgery. Pakistan Journal of Ophthalmology. 2010 Mar 31;26(1).	Excluded
171	BERNTH-PETERSEN PE. Outcome of cataract surgery I: A prospective, observational study. Acta ophthalmologica. 1982 Apr;60(2):235-42.	Excluded
172	Addisu Z, Solomon B. Patients' preoperative expectation and outcome of cataract surgery at jimma university specialized hospital-department of ophthalmology. Ethiopian journal of health sciences. 2011;21(1):47-56.	Excluded
173	Ji MJ, Kim MS, Lee SJ, Han SB. Evaluation of Visual Outcome after Cataract Surgery in Patients Aged 85 Years or Older. Journal of the Korean Ophthalmological Society. 2016 Feb 1;57(2):214-20.	Excluded
174	Ezegwui IR, Ajewole J. Monitoring cataract surgical outcome in a Nigerian mission hospital. International ophthalmology. 2009 Feb;29:7-9.	Excluded
175	Murthy GV, Gupta SK, Talwar D. Assessment of cataract surgery in rural India. Visual acuity outcome. Acta ophthalmologica scandinavica. 1996 Feb;74(1):60-3.	Excluded
176	Elhusseiny AM, Soliman MK, Shakarchi AF, Fouad YA, Yang YC, Sallam AB. Visual outcomes and complications of combined vs sequential cataract surgery and pars plana vitrectomy: multicenter database study. Journal of Cataract & Refractive Surgery. 2023 Feb 1;49(2):142-7.	Excluded
177	Agervi P, Kugelberg U, Kugelberg M, Zetterström C. Refractive and visual outcome of paediatric cataract surgery in the Ukraine. Acta Ophthalmologica Scandinavica. 2006 Oct;84(5):674-8.	Excluded
178	Qi Y, Zhang YF, Zhu Y, Wan MG, Du SS, Yue ZZ. Prognostic factors for visual outcome in traumatic cataract patients. Journal of ophthalmology. 2016;2016(1):1748583.	Excluded
179	Kandel RP, Sapkota YD, Sherchan A, Sharma MK, Aghajanian J, Bassett KL. Cataract surgical outcome and predictors of outcome in Lumbini Zone and Chitwan District of Nepal. Ophthalmic epidemiology. 2010 Oct 1;17(5):276-81.	Excluded

180	Rey A, Jürgens I, Maseras X, Dyrda A, Pera P, Morilla A. Visual outcome and complications of cataract extraction after pars plana vitrectomy. Clinical Ophthalmology. 2018 May 25:989-94.	Excluded
181	Rey A, Jürgens I, Maseras X, Dyrda A, Pera P, Morilla A. Visual outcome and complications of cataract extraction after pars plana vitrectomy. Clinical Ophthalmology. 2018 May 25:989-94.	Excluded
182	Lundström M, Stenevi U, Thorburn W, Roos P. Catquest questionnaire for use in cataract surgery care: assessment of surgical outcomes. Journal of Cataract & Refractive Surgery. 1998 Jul 1;24(7):968-74.	Excluded
183	Rabiu MM, Jenf M, Fituri S, Choudhury A, Agbabiaka I, Mousa A. Prevalence and causes of visual impairment and blindness, cataract surgical coverage and outcomes of cataract surgery in Libya. Ophthalmic epidemiology. 2013 Feb 1;20(1):26-32.	Excluded
184	, Bensaid P, Resnikoff S, Seini K, Landreau N, Paugam JM, Nagot N, Mura T, Serrand C, Villain M, Daien V. Outcomes of cataract surgery performed by non-physician cataract surgeons in remote North Cameroon. British Journal of Ophthalmology. 2019 Aug 1;103(8):1042-7.	Excluded
185	Lewallen S. The importance of biometry to cataract outcomes in a surgical unit in Africa. Ophthalmic Epidemiology. 2010 Aug 1;17(4):196-202.	Excluded
186	Courtright P, Metcalfe N, Hoechsmann A, Chirambo M, Lewallen S, Barrows J, Witte C, Kanjaloti S, Mtambo O, Chipeta T, Mekisini G. Cataract surgical coverage and outcome of cataract surgery in a rural district in Malawi. Canadian journal of ophthalmology. 2004 Feb 1;39(1):25-30.	Excluded
187	Chirambo M, Lewallen S, Barrows J, Witte C, Kanjaloti S, Mtambo O, Chipeta T, Mekisini G. Cataract surgical coverage and outcome of cataract surgery in a rural district in Malawi. Canadian journal of ophthalmology. 2004 Feb 1;39(1):25-30.	Excluded
188	Prasad M, Daigavane S, Kalode V. Visual outcome after cataract surgery in rural hospital of Wardha district: a prospective study. Journal of Clinical and Diagnostic Research. 2020 Feb 1;14(2):4-6.	Excluded
189	Meltzer ME, Congdon N, Kymes SM, Yan X, Lansingh VC, Sisay A, Müller A, Chan VF, Jin L, Karumanchi SM, Guan C. Cost and expected visual effect of interventions to improve follow-up after cataract surgery: prospective review of early cataract outcomes and grading (PRECOG) study. JAMA ophthalmology. 2017 Feb 1;135(2):85-94.	Excluded
190	Meltzer ME, Congdon N, Kymes SM, Yan X, Lansingh VC, Sisay A, Müller A, Chan VF, Jin L, Karumanchi SM, Guan C. Cost and expected visual effect of interventions to improve follow-up after cataract surgery: prospective review of early cataract outcomes	Excluded

	and grading (PRECOG) study. JAMA ophthalmology. 2017 Feb 1;135(2):85-94.	
191	Lewallen S. The importance of biometry to cataract outcomes in a surgical unit in Africa. Ophthalmic Epidemiology. 2010 Aug 1;17(4):196-202.	Excluded
192	Jackson H, Garway-Heath D, Rosen P, Bird AC, Tuft SJ. Outcome of cataract surgery in patients with retinitis pigmentosa. British Journal of Ophthalmology. 2001 Aug 1;85(8):936-8.	Excluded
193	Bilbao A, Vidal S, de Larrea NF, Díaz V, Gracia J, IRYSS-Cataract Group. Factors affecting cataract surgery complications and their effect on the postoperative outcome. Canadian Journal of Ophthalmology. 2014 Feb 1;49(1):72-9.	Excluded
194	Lewallen S. The importance of biometry to cataract outcomes in a surgical unit in Africa. Ophthalmic Epidemiology. 2010 Aug 1;17(4):196-202.	Excluded
195	Heath D, Rosen P, Bird AC, Tuft SJ. Outcome of cataract surgery in patients with retinitis pigmentosa. British Journal of Ophthalmology. 2001 Aug 1;85(8):936-8.	Excluded
196	Ezegwui IR, Ajewole J. Monitoring cataract surgical outcome in a Nigerian mission hospital. International ophthalmology. 2009 Feb;29:7-9.	Excluded
197	Briesen S, Roberts H, Lewallen S. The importance of biometry to cataract outcomes in a surgical unit in Africa. Ophthalmic Epidemiology. 2010 Aug 1;17(4):196-202.	Excluded
198	Dhalla KA, Jecha J, Kapesa I, Odouard C, Murdoch I. Results and safety profile of trainee cataract surgeons in a community setting in East Africa. Indian journal of ophthalmology. 2016 Nov 1;64(11):818-21.	Excluded
199	Zitha AJ, Rampersad N. Cataract surgery outcomes: comparison of the extracapsular cataract extraction and manual small incision cataract surgery techniques. African Health Sciences. 2022 Apr 29;22(1):619-29	Excluded
200	Bekibele CO. A comparative evaluation of outcome of cataract surgery at Ago-Iwoye, Ogun State. Nigerian Journal of Surgical Research. 2004;6(1-2).	Excluded
201	Ugalahi MO, Uchendu OC, Ugalahi LO. Preoperative visual acuity of cataract patients at a tertiary hospital in sub-Saharan Africa: a 10-year review. Therapeutic Advances in Ophthalmology. 2019 Nov;11:2515841419886451.	Excluded
